# Comparative efficacy of pharmacological and nonpharmacological treatments for chronic idiopathic constipation in China: a Bayesian network meta-analysis

**DOI:** 10.1186/s12906-019-2741-z

**Published:** 2019-11-14

**Authors:** Qingyang Shi, Lizi Tan, Chunxiang Liu, Huijun Wang, Junhua Zhang, Hui Wang, Jingbo Zhai

**Affiliations:** 1 Tianjin University of Traditional Chinese Medicine, Beihua south road, Jinghai district, Tianjin, 301617 China; 2 Evidence-based Medicine Center, Tianjin University of Traditional Chinese Medicine, Tianjin, 301617 China

## Abstract

**Background:**

To provide evidence for medical management of chronic idiopathic constipation (CIC) in China based on comparisons of all clinical practical interventions using Bayesian network meta-analysis.

**Methods:**

We conducted a systematic literature review by searching PubMed, Embase, Cochrane Central, the China National Knowledge Infrastructure (CNKI), and the Wanfang Database (inception to May 2019) for randomized controlled trials (RCTs) for CIC in Chinese people. Only RCTs that recruited participants aged over 18 and diagnosed with CIC by the Rome II, III or IV criteria were included. We used three outcomes to examine efficacy. The risk ratio (RR) of the responder rate, based on ≥3 spontaneous bowel movements (SBMs) per week after treatment, was the primary outcome, and the SBM count per week and the Bristol score (BS) were secondary outcomes. In addition, adverse effects (AEs) were also considered a secondary outcome to evaluate safety. We conducted Bayesian network meta-analysis with random effects, and the RR or mean difference with its 95% credible interval was calculated. In addition, we ranked all treatments via their cumulative curves (SUCRA) and assessed the quality of evidence according to the GRADE criteria.

**Results:**

We included a total of 42 trials (6820 participants) of 20 grouped interventions that included pharmacological and nonpharmacological treatments. For the primary outcome, fourteen interventions were significantly better than placebo, and Probiotics plus Mosapride (PB + MP) appeared superior to others (GRADE quality of evidence: Moderate to Low), followed by Prucalopride (PP) (High to Low) and Electroacupuncture (EA) (High to Low). For SBM, Compound sodium bicarbonate suppository (CSBS) appeared to be best, with an SUCRA value of 90% (High to Low). For BS, Lactulose plus Probiotics (LT + PB) was superior to others (Moderate to Low), followed by Polyethylene glycol (PEG) (High to Moderate). Although all interventions appeared non-significant when compared with placebo in terms of adverse effects, Lactulose plus Mosapride showed greater risk than others on ranking probability.

**Conclusions:**

Given the GRADE assessment, PB + MP, PP and EA may be the priory options with moderate certainty in the quality of evidence for the primary outcome. For SBM, a CSBS may be the best option with moderate certainty in the quality of evidence. For BS, PEG may be the priory option with high certainty in the quality of evidence. However, due to a lack of high certainty in the quality of evidence, caution is needed when recommending the interventions. Because of the limitations, an increased number of trials are required for more accurate results.

## Background

Chronic idiopathic constipation (CIC), also known as functional constipation, is identified by predominant symptoms such as incomplete, difficult and infrequent defecation (typically less than 3 times a week) [1, 2]. Some patients may have abdominal bloating and hard consistency of stools [3]. Today, the Rome IV symptom criteria are widely used to diagnose CIC [1, 4]. The estimated pooled global prevalence of chronic idiopathic constipation is 14% [5], and 33% of adults older than 60 years suffer from constipation symptoms [2]. Being one of the most common gastrointestinal disorders [6], CIC contributes nearly 1.6 million ambulatory visits to health care institutions annually in the United States of America and is the 7th leading physician diagnosis in ambulatory settings [7]. The prevalence of CIC among Chinese adults is 4%~ 6% [8]. As reported in two review articles, 18.1% of the elderly Chinese population and more females than males suffer from CIC, which indicates a female and age predominance in this disorder [9, 10]. It causes a significant impact on an individual’s quality of life and results in a heavy financial burden [10].

Dietary fiber supplements, osmotic laxatives and serotonin 5-HT4 receptor agonists are generally recommended as conventional medications [8, 11]. However, approximately 50% of patients are thought to be unsatisfied with their treatments, especially with fiber supplements and laxatives (neither stimulant nor osmotic), and the main complaints are safety concerns and insufficient efficacy [12]. One network meta-analysis provides evidence that Bisacodyl may show a better performance on changing the number of spontaneous bowel movements (SBMs) per week in CIC [12]. However, due to different medical management environments, practitioners dealing with ethnic Chinese groups are facing a challenge with choosing the appropriate therapeutic method. Several intestinal secretagogues, such as lubiprostone and linaclotide, have not been approved for marketing in China, and traditional Chinese medicine (TCM) therapies, including herbal decoction and acupuncture, are recommended as complementary therapies and are commonly used in clinical practice [8]. Therefore, we hope to provide evidence for the medical management of CIC for the whole ethnic Chinese population based on the efficacy and safety of all clinical practical interventions.

## Method

We conducted this systematic review and network meta-analysis following guidance from the Cochrane Handbook for Systematic Reviews of Interventions [13]. The Preferred Reporting Items for Systematic Reviews and Meta-Analyses guidelines were also followed [14].

We followed an a priori designed protocol registered on PROSPERO with ID CRD42018114327. We also performed some protocol amendments for the primary outcome whose details are shown in the Additional file 1: File S1. A thorough database search was performed by two investigators independently, using PubMed, Embase, Cochrane Central, the China National Knowledge Infrastructure (CNKI), and the Wanfang Database for the ethnic Chinese group for the treatment of CIC from inception to May 2019 without language restriction. All the studies included were identified with the following search strings: “Chronic Idiopathic Constipation”, “Functional Constipation” and “randomized controlled study”. The complete search strategy is shown in the Additional file 1: File S2. Only randomized controlled trials (RCTs) that recruited participants aged over 18 and diagnosed with CIC by the Rome II, III or IV criteria were included. We excluded studies that applied inaccurate RCT methodologies or quasi-RCTs. In addition, when filtering the Chinese studies, we only included those from the CNKI and Wanfang databases indexed by the *Chinese Science Citation Database* (CSCD) and the *Chinese Core Journals by Peking University* (PKU).

Studies included in this review reported their final efficacy in different ways, which made it difficult to synthesize the final results. Thus, we analyzed the different end points and extracted the same kind of information from the different outcome results as a unified outcome indicator that could be evaluated. The primary end point in this review was the responder rate, based on ≥3 SBMs/week after treatment. We applied some continuous variables with important clinical significance to address the secondary end points: the change from baseline in the number of SBMs/week (SBMs), the Bristol score (BS) and the number of adverse effects (AEs).

Two investigators independently performed the data extraction process (Shi and Tan) for the primary and secondary end points. We also extracted characteristics of the study and participant characteristics, such as age, proportion of females and number of enrollments in every study, type of intervention, duration of treatment, course of disease, whether the study was a multicenter trial, outcome measures, whether there was intention-to-treat (ITT), and the number of participants lost to follow-up.

Finally, following the Cochrane Collaboration guidelines [13], two investigators independently assessed the study quality. Nine domains were considered: random sequence generation (selection bias), allocation concealment (selection bias), blinding of participants and personnel (performance bias), blinding of outcome assessment (detection bias), incomplete outcome data (attrition bias), selective reporting (reporting bias), whether there was ITT, number of participants lost to follow-up, and other bias.

We used a Bayesian network meta-analysis with a random effects model to synthesize the data for each outcome [15]. Based on the consistency model between direct and indirect evidence, we combined the relative effects for all possible comparisons (direct and indirect). We assumed a vague prior for the between-study heterogeneity with uniform distribution. The models were optimized, and estimations were obtained using Markov chain Monte Carlo (MCMC) methods, with weighting for sample size [16]. The convergence of the MCMC model was assessed using the Brooks-Gelman-Rubin method [17]. We applied the (Log) risk ratio (RR) to describe categorical variables and the mean difference (MD) for continuous variables. The I-square statistical method, which represents the proportion of variance attributable to study heterogeneity, was performed to assess heterogeneity. Potential inconsistent loops were detected by node-splitting analysis, and then we presented inconsistency *P*-values for each comparison.

The analyses of primary outcome, the risk ratio of the responders rate, were performed under the model assumption of a binomial distribution likelihood and a “log” link function, which were used to compute the posterior distribution of the effect of each intervention compared with the placebo (or compared with each other). We presented these results as the relative effect sizes of the two interventions with the median of the posterior distribution and 95% credible intervals. We analyzed the secondary outcomes in the same way, except for a normal distribution likelihood and an “Identify” link function rather than a binomial distribution for continuous variables. We ranked treatments by their posterior probability by calculating the Surface Under the Cumulative Ranking (SUCRA) curve values and reported their median ranks with 95% credible intervals in forest plots [18]. Comparison-adjusted funnel plots were obtained with the specific ranking order to detect small-study effects and publication bias.

To detect potential heterogeneity sources and lower the bias from confounding, we conducted univariate meta-regressions. Twelve regressors were considered: duration of treatment, course of disease, proportion of women, sample size, age, whether ITT analysis was reported, whether the study quality was low, whether randomization sequences were generated, whether allocation concealment was performed, whether the study was double-blind, whether the trial was a multicenter trial, whether participants were lost to follow-up. We conducted a multiple imputation to handle missing data with the random forest method [19]. Then, we reported the posterior median of the interaction parameters and their 95% credible intervals for all regressors. In addition, we conducted sensitive analyses with different priors for between-study heterogeneity and used the “power adjust” argument to downweight studies, which applies a variance inflation to the likelihood. We specified the weight for each study using the Cochrane bias tool, which allocates a small weight to low quality studies.

Furthermore, to rate the quality of evidence, we assessed the direct, indirect and mixed estimates of all comparisons in accordance with GRADE criteria [20]. In particular, we used the approach for indirect and network evidence by Puhan et al. [21] and its complemented version by Brignardello-Petersen et al. [22] We evaluated the direct estimates following the original five GRADE criteria (Risk of bias; Inconsistency; Indirectness; Imprecision; Publication bias) but removed the “Imprecision” criterion in this step. Instead, we applied this criterion to the network estimate assessment as recommended by Brignardello-Petersen et al.

All computations were performed using the R (V.3.5.1) package gemtc (V.0.8.1) [23], along with the Markov chain Monte Carlo engine JAGS (V.3.4.0). The risk of bias graph was generated by Cochrane RevMan 5.3 [24].

## Result

### Literature review

**Fig. 1:**
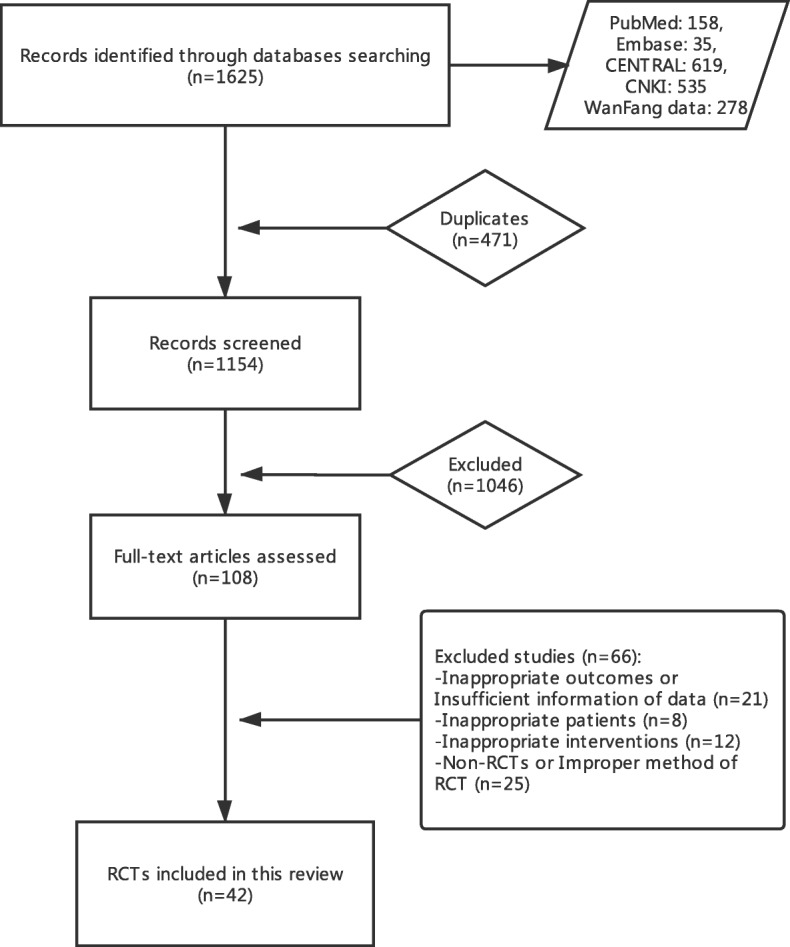
Flowchart of study selection and identification

Of the 1625 citations identified, 812 were in English, and 813 were in Chinese. We identified 108 citations for full-text-appraisal review after screening for incorrect titles and abstracts. Through the review process, we excluded studies with no end point reported in terms of responder rate or SBM, inappropriate interventions, or inappropriate patients and that were not RCTs or that applied improper RCT methods. Finally, 42 studies were included in our quantitative analysis review [25–66]. Figure 1 illustrates the screening process, and the Additional file 1: Table S1 reveals the study characteristics.

The 42 included trials consisted of a total of 91 treatment arms and 6820 participants. All studies were parallel controlled designs, among which three studies were designed with three arms. The average age of all participants was 52, and the average gender ratio was 67.4%.

Eleven types of intervention were investigated in this study. All treatments were grouped into 20 categories: 15 single interventions and 5 combinations of interventions. The 15 single interventions were as follows: Mosapride (MP), Probiotics (PB), Lactulose (LT), Polyethylene glycol (PEG), Soluble fiber (SF), Insoluble fiber (IF), Prucalopride (PP), Electroacupuncture (EA), Chinese herbal decoction (CHD), Stimulant laxatives (SL), Hemp seed pill (HS), Compound sodium bicarbonate suppository (CSBS), Recreation therapy (RT), Acupoint catgut-embedding (ACE) and placebo.

**Fig. 2:**
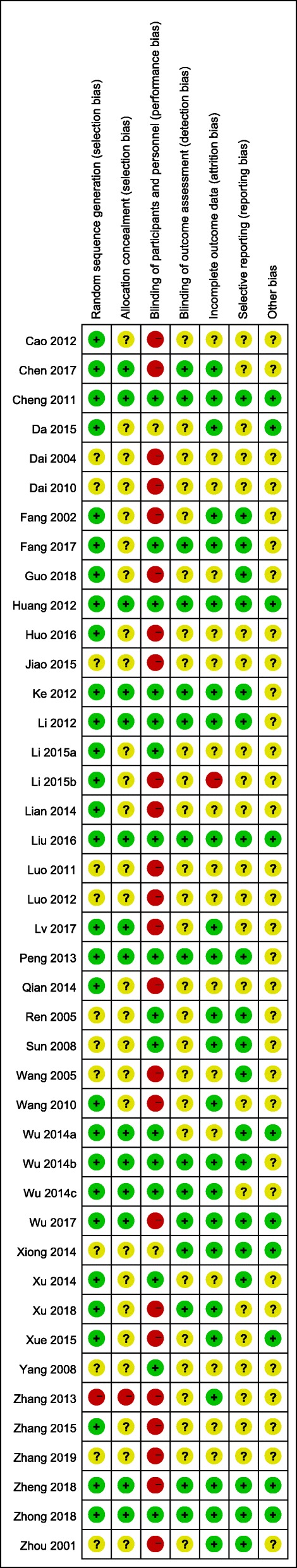
Risk of bias summary

Study quality is summarized in Fig. 2 and Additional file 1: Table S2. Thirty-six of the studies were of high to moderate quality, while six were of low quality. Among them, 69% reported specific random sequence generation methods, 38% were double-blind studies, 36% were multicenter trials, and 45% applied ITT analysis.

### Network meta-analysis

#### Risk ratio of the primary outcome

**Fig. 3:**
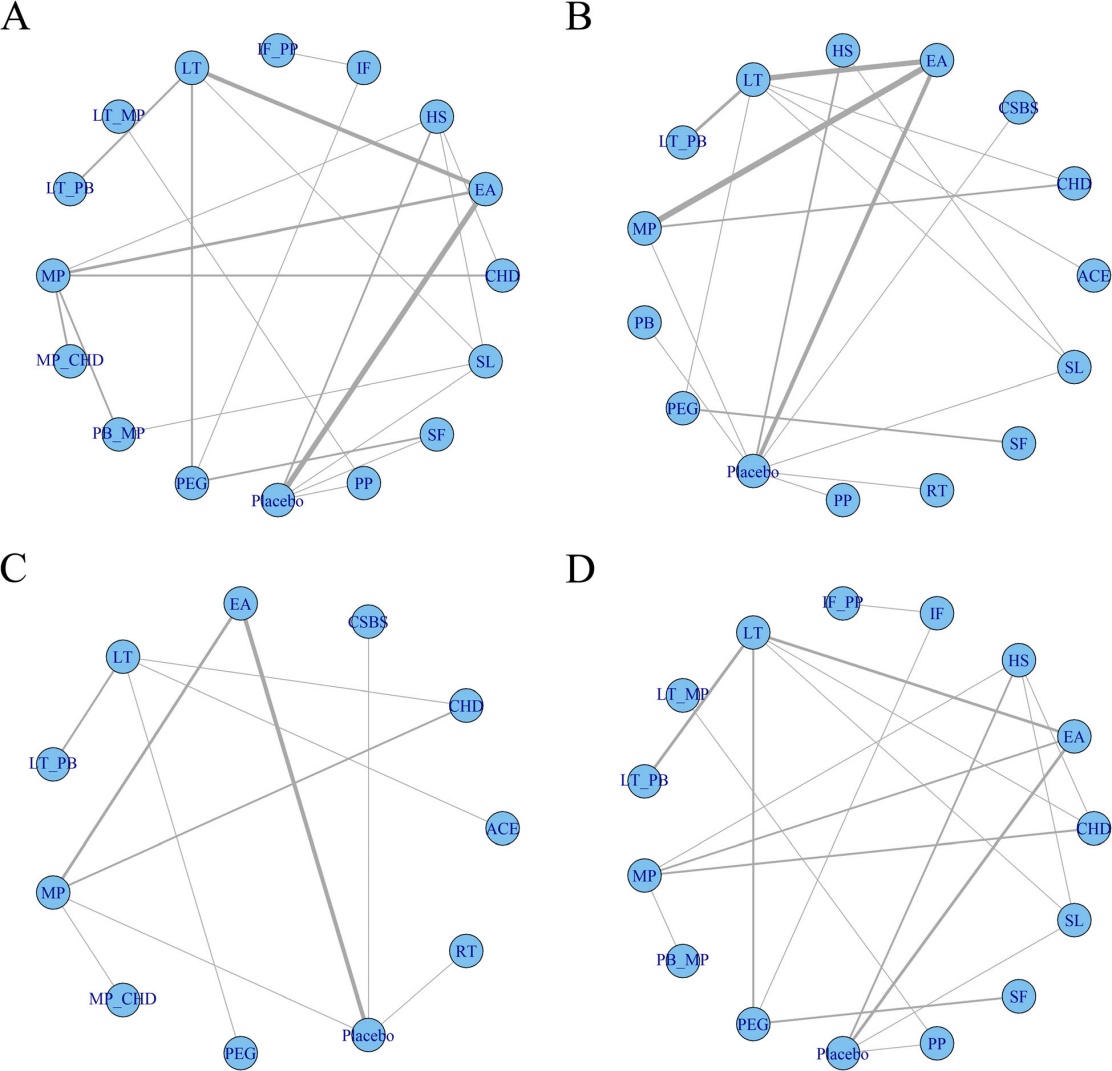
Network plot. **a** Network plot of the primary outcome. The primary outcome is the responder rate, based on ≥3 SBMs/week after treatment. **b** Network plot of the SBM count. **c**: Network plot of the BS. **d**: Network plot of adverse effects

**Fig. 4:**
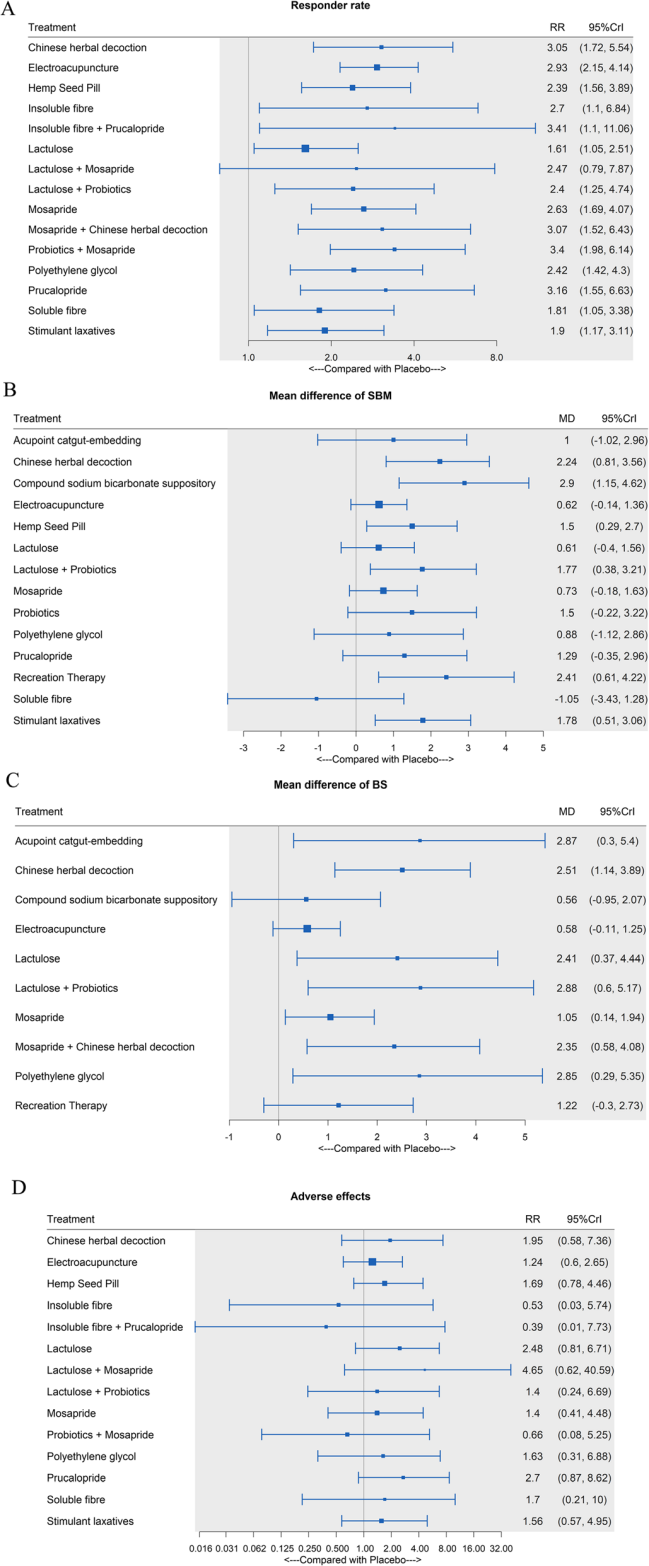
Forest plot. **a** The risk ratio of the responder rate. **b** The mean difference of the SBM count. **c**: The mean difference of the BS. **d**: The risk ratio of adverse effects

**Fig. 5:**
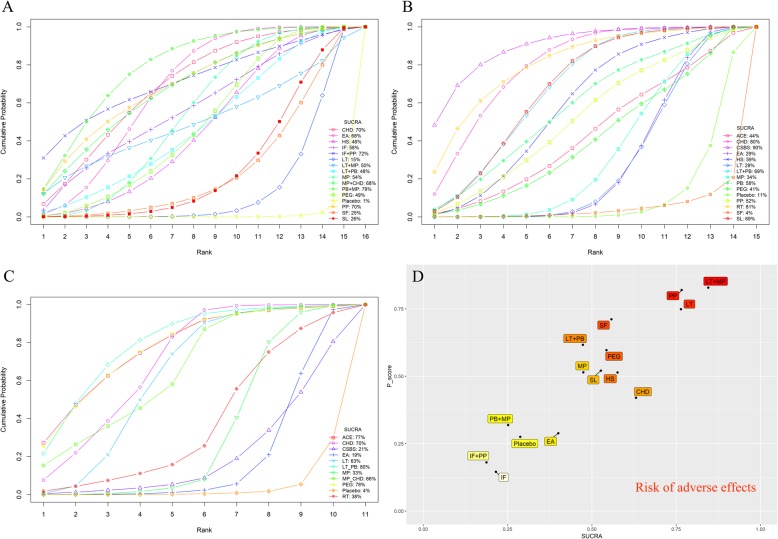
Cumulative probability curves and SUCRA values. **a** The risk ratio of the responder rate. **b** The mean difference of the SBM count. **c**: The mean difference of the BS. **d**: Biplot of SUCRA values and P-scores of adverse effects

**Fig. 6:**
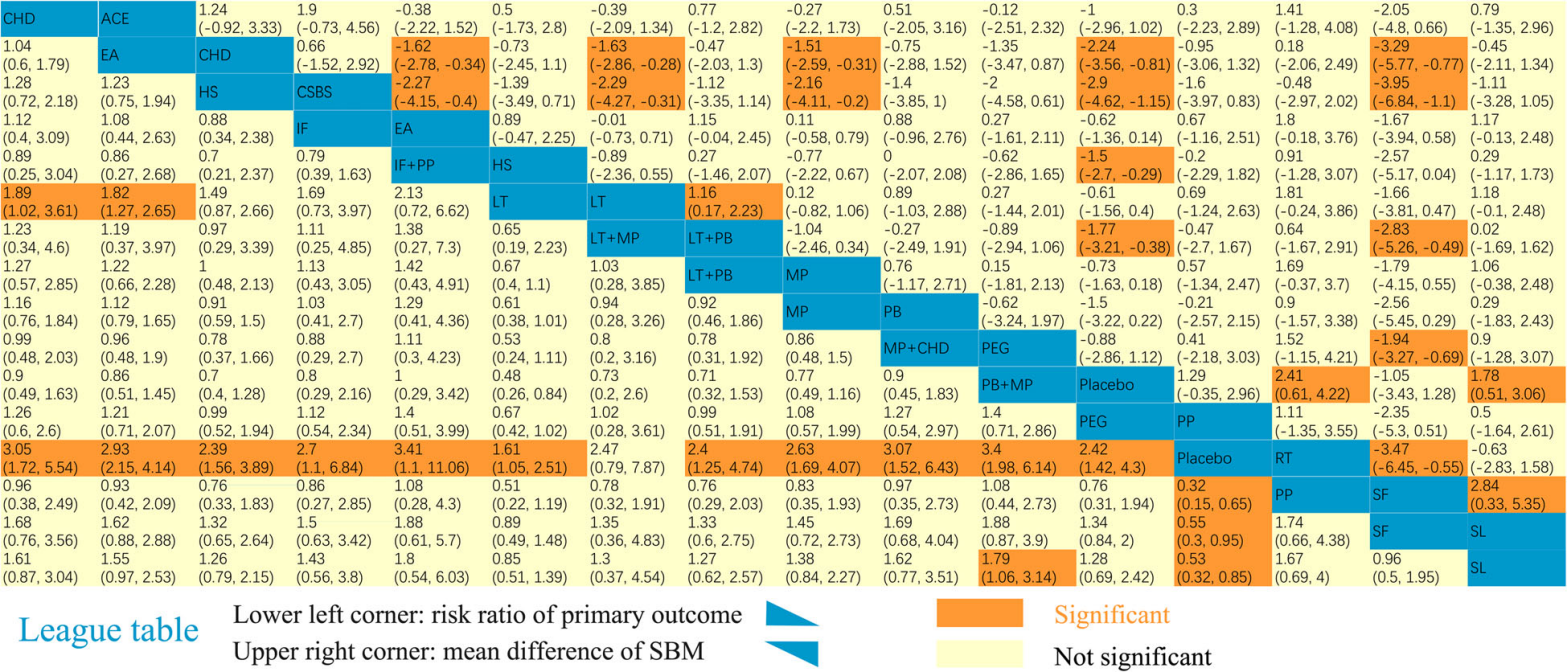
League table of network estimates. Lower left corner: The risk ratio of the responder rate Upper right corner: The mean difference of the SBM count.

Fifteen interventions as well as placebo were included. The network plot is shown in Fig. 3a. Except for LT + MP, all the interventions showed significant differences compared with placebo (Fig. 4a). In the SUCRA graph (Fig. 5a), PB + MP was superior to the others, followed by IF+PP, PP and CHD (SUCRA: 79, 72, 70, 70%, respectively). All treatments are compared with each other in the league table in the lower-left corner of Fig. 6.

To illustrate the inconsistency in closed loops, we performed node-splitting analysis to obtain effect sizes for all possible comparisons (direct and indirect), as shown in Additional file 2: Page S1. Four comparisons had significant differences between the direct and indirect results: placebo vs EA, PEG vs LT, SF vs PEG and SF vs placebo (*P*-values: 0.045, 0.033, 0.033, 0.034, respectively).

**Fig. 7:**
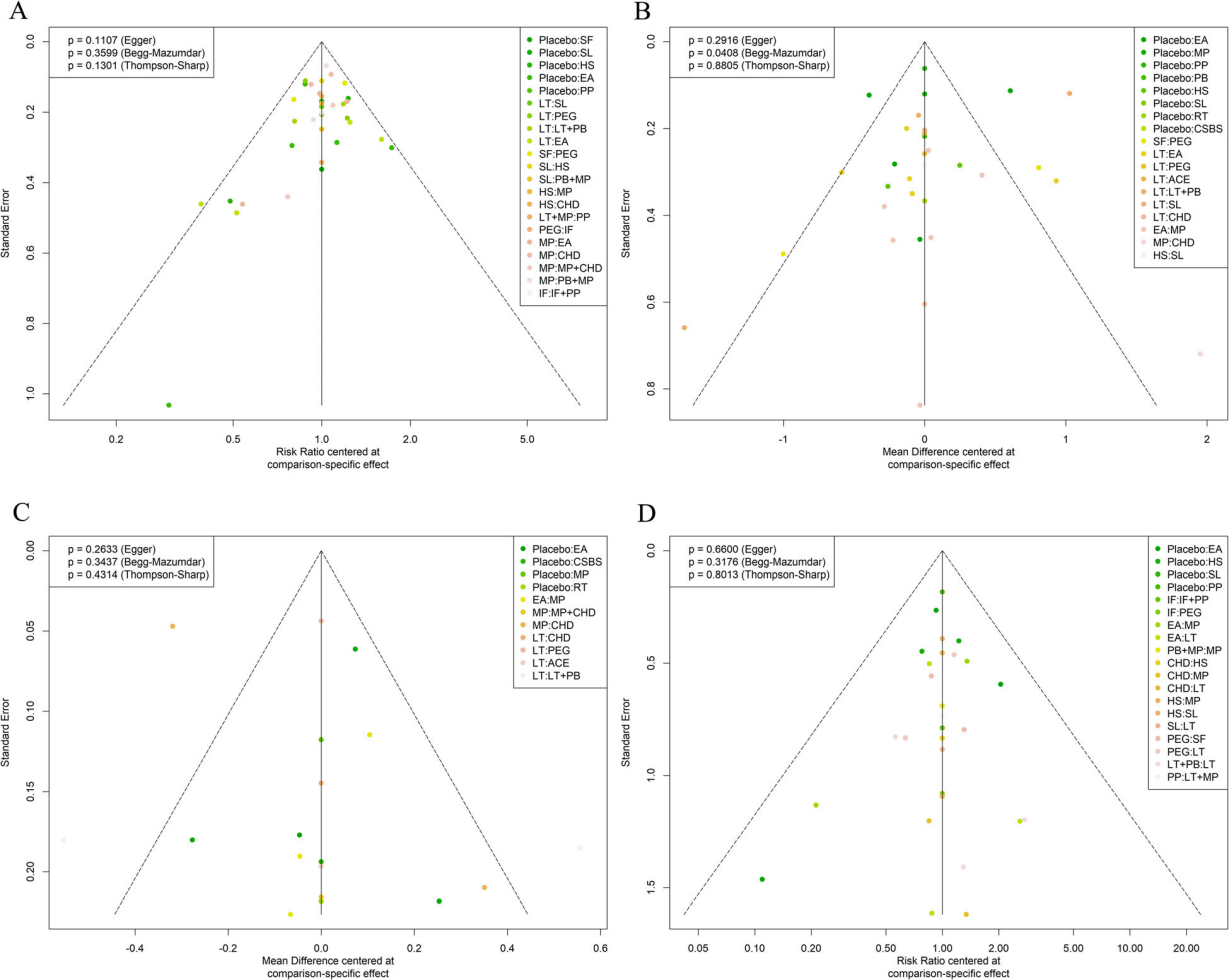
Funnel plot. **a** The risk ratio of the responder rate. **b** The mean difference of the SBM count. **c** The mean difference of the BS. **d** The risk ratio of adverse effects

In addition, we conducted a meta-regression and sensitivity analysis for primary outcome. The meta-regression results showed that two covariates, age and loss to follow-up, had significant coefficients (β = − 2.56, 95%CrI[− 4.56, − 0.7]; − 1.01, [− 2.1, − 0.045], respectively) (Additional file 1: Table S3). Then, after adjusting for the covariates, the SUCRA values of some interventions changed, as shown in the Additional file 1: Table S4. In the sensitivity analysis, the power-adjusted model showed that PB + MP, which had the highest SUCRA value in the original model, had a decreased SUCRA value (67%), and instead PP had the highest value (SUCRA: 75%). The other models, with alternative priors, showed no change. The funnel plot showed no significant asymmetry with Egger’s or Begg’s test (Fig. 7a).

### Secondary outcomes

#### Spontaneous bowl movement

Fourteen interventions as well as placebo were included. The network plot is shown in Fig. 3b. Compared with the placebo, six interventions (CHD, CSBS, HS, LT + PB, RT and SL) showed significant differences (Fig. 4b). Among them, CSBS was superior to the others, followed by RT and CHD (SUCRA: 90, 81, 80%, respectively) (Fig. 5b). All treatments are compared with each other in the league table in the upper-right corner of Fig. 6. In the node-splitting analysis, no significant difference between direct and indirect results was detected (Additional file 2: Page S2). No significant coefficient was found in the meta-regression (Additional file 1: Table S5). In the funnel plot (Fig. 7b), a significant asymmetry was shown with Begg’s test (*P* = 0.04) but not with Egger’s test (*P* = 0.29).

#### Bristol stool

Ten interventions as well as placebo were included. The network plot is shown in Fig. 3c. Seven interventions (ACE, CHD, LT, LT + PB, MP, MP + CHD and PEG) were significantly better than placebo (Fig. 4c). LT + PB was superior to the others, followed by PEG and ACE (SUCRA: 80, 78, 77%, respectively) (Fig. 5c). No significant asymmetry was shown in the funnel plot (Fig. 7c).

#### Adverse events

Fourteen interventions as well as placebo were included. Figure 3d shows the network plot. No intervention was significantly different from placebo (Fig. 4d). However, the interventions showed different rankings of adverse effects risks in their posterior probability. To detect the different risks of the interventions, in addition to Bayesian SUCRA values, we also used frequentist P-scores to illustrate their ranking. Three interventions (LT + MP, PP and LT) were shown to have a high risk of adverse effects, and IF had the lowest risk (Fig. 5d). No significant asymmetry was found in the funnel plot (Fig. 7d).

#### GRADE assessment

We assessed the quality of evidence for all outcomes with all comparisons (Additional file 1: Table 6-8) and summarized them in Table 1. The quality of evidence was grouped into three categories with different certainties (high certainty, moderate certainty and low certainty). Among them, low certainty evidence and interventions were not recommended, which means that the effect estimates of this kind of evidence are likely to be substantially different from the true effect. The interventions were grouped into two categories according to the SUCRA values (SUCRA > = 80% for group 1; others for group 2). In the results, we found that three interventions (PB + MP, PP and EA) could be recommended with moderate certainty for the primary outcome, but they were not the best interventions with sufficient confidence due to the SUCRA value. For SBM, CSBS could be recommended with moderate certainty and a high SUCRA value, followed by RT. For BS, PEG could be recommended with high certainty.

**Table 1:** GRADE Assessment

Outcomes	Certainty on the evidence	Classification	Intervention	SUCRA	GRADE assessment
Primary outcome	High Certainty (High to Moderate quality evidence)	–	None	–	–
	Moderate Certainty (High to Low or Moderate to Low quality evidence)	Group 1 Amongst the best interventions	None	–	–
		Group 2 Inferior to the best interventions	PB + MP	79%	Moderate to Low
			PP	70%	High to Low
			EA	68%	High to Low
	Low Certainty (High to Very low or Moderate to Very low or Low to Very low quality evidence)	Group 1 Amongst the best interventions	None	–	–
		Group 2 Inferior to the best interventions	IF+PP	72%	Moderate to Very low
			CHD	70%	Low to Very low
			MP + CHD	68%	Low to Very low
Spontaneous bowel movements count	High Certainty (High to Moderate quality evidence)	–	None	–	–
	Moderate Certainty (High to Low or Moderate to Low quality evidence)	Group 1 Amongst the best interventions	CSBS	90%	High to Low
			RT	81%	High to Low
		Group 2 Inferior to the best interventions	None	–	–
	Low Certainty (High to Very low or Moderate to Very low or Low to Very low quality evidence)	Group 1 Amongst the best interventions	CHD	80%	Moderate to Very low
		Group 2 Inferior to the best interventions	SL	69%	High to Very low
			LT + PB	69%	Low to Very low
Bristol score	High Certainty (High to Moderate quality evidence)	Group 1 Amongst the best interventions	None	–	–
		Group 2 Inferior to the best interventions	PEG	78%	High to Moderate
	Moderate Certainty (High to Low or Moderate to Low quality evidence)	Group 1 Amongst the best interventions	LT + PB	80%	Moderate to Low
		Group 2 Inferior to the best interventions	None	–	–
	Low Certainty (High to Very low or Moderate to Very low or Low to Very low quality evidence)	Group 1 Amongst the best interventions	None	–	–
		Group 2 Inferior to the best interventions	ACE	77%	High to Very low
			CHD	70%	High to Very low

## Discussion

### Summary of evidence

In this network meta-analysis, we included 42 studies with 6820 participants and 20 grouped interventions. Among them, 30 studies reported the primary outcome, 28 reported SBM, 17 reported BS and 24 reported AE.

For the risk ratio of the primary outcome, we found that 14 interventions were significantly better than placebo, and PB + MP had the best efficacy with a SUCRA value of 79%. In addition, after adjusting for covariates, PB + MP was still the best intervention among the 14. However, in the power-adjusted model for study quality, we found that PP was the best intervention, while PB + MP had a decreased SUCRA value. In the results for SBM, six interventions were significantly better than placebo, and CSBS was the best with a SUCRA value of 90%. For BS, seven interventions were significantly better than placebo, and LT + PB was superior to the others. To summarize these three outcomes, we plotted their median ranks with the corresponding 95%CrIs in a forest plot (Additional file 2: Page S3). We found that CHD had a comprehensive efficacy (median rank: RR: 5 [1, 13]; SBM: 3 [1, 9]; BS: 4 [1, 7]), although it did not rank first for any of the three outcomes. For adverse effects, even though all interventions showed no significant difference compared with placebo, they had different risks in the ranking biplot. LT + MP, PP and LT showed more risks than others. Further attention should therefore be paid to the balance between safety and clinical benefits of these interventions.

According to the quality of evidence obtained with the GRADE assessments, we recommend PB + MP, PP and EA as prior options for primary outcome; CSBS and RT for SBM; PEG and LT + PB for BS. However, except for PEG, with a high certainty in the quality of evidence, all the others had moderate certainty. Thus, caution is needed to interpret the results.

### Strengths and limitations

Network meta-analysis allows us to make net comparisons across different interventions and to estimate the efficacies of pharmacotherapies and nonpharmacological therapies. Through direct and indirect efficacy comparisons of the end point indicators of different interventions, we are able to make relatively appropriate recommendations for different patient groups. This review only included RCTs that recruited ethnic Chinese people, which strengthens the accuracy of extrapolation for the studies. This review can provide assistance in CIC management guideline formulation for the Chinese region. The validity of this study is well assured. Of the trials included, 36% were multicenter studies, which enhanced the external validity. Regarding internal validity, we found that 69% of the studies reported a specific random sequence generation method, 52% reported no or less than 5% loss to follow-up, and half of the studies performed ITT analysis, which makes the internal validity more convincing. Moreover, for better quality control, we only included those studies indexed by the CSCD and PKU when filtering Chinese studies. In addition, to lower the bias from low quality studies, we conducted a power-adjusted model to detect their robustness. Finally, we performed meta-regressions to detect the bias from confounding variables and presented the changed SUCRA values after adjusting for the significant coefficients.

There are, however, some limitations in the results. Bias in the evaluation of some interventions may be generated from not reporting the primary end point as the responder rate in the corresponding studies. Instead, these studies only reported the SBM or BS, and so a larger sample of studies to address this issue more clearly. In acupuncture studies, we performed data merging on similar electroacupuncture interventions (such as different current intensities) to more precisely appraise acupuncture interventions as a whole in comparison with other interventions. However, this may have resulted in a larger standard deviation, which could lead to imprecision of the results.

In the node-splitting analysis for the primary outcome, we detected four comparisons with significant differences between direct and indirect results. However, the inconsistency had a borderline significant difference, which means that it had little effect on the pooled results. We considered it a downgrading factor in the GRADE assessment for network estimates.

In the meta-regression, we found that “age” and “lost to follow-up” had significant coefficients. Thus, these two covariates may have impacted the pooled results, and after adjusting for them, we found some changed SUCRA values. The best intervention, PB + MP, maintained its ranking, which means that it has a robust result in the meta-regression. However, in the power-adjusted model, PB + MP had a lower SUCRA value, resulting in PP and IF becoming the top two interventions. This means that there could also be a potential impact on the study quality, although we partly controlled for this before. Therefore, the study quality was considered a downgrading factor for direct evidence in the GRADE assessment.

Less than half of the included studies reported conducting ITT analysis while also not providing information about follow-up, although we did not find a significant coefficient after meta-regression for “whether ITT analysis was reported”. Therefore, even though we adjusted for “lost to follow-up” in the meta-regression, bias still cannot be totally eliminated for the studies that did not report information on this. Only 38% of the trials were double-blinded, which could bring the placebo effect, and exaggeration of the results may be anticipated.

In addition, bias in the appraisal of multiple interventions may result from the limited number of studies included due to our restriction on investigating ethnic Chinese people only. A confirmation of no efficacy of some interventions in the secondary outcomes of SBM and BS requires more RCTs for a narrower credible interval and more convincing appraisal results; for now, we can merely suggest that these interventions have an uncertain efficacy.

## Conclusion

In conclusion, this review provides evidence for the treatment of Chinese CIC patients. Given the GRADE assessment results, we recommended PB + MP, PP and EA with moderate certainty in the quality of evidence for primary outcome. CSBS was recommended with moderate certainty for SBM, and PEG was recommended with high certainty for BS. However, except for CSBS, all the others should not be considered as the best option due to their SUCRA values. In addition, except for PEG, all the others had moderate certainty in the quality of evidence, which means that caution is needed when using these interventions. Further assessments are needed for a more accurate result due to the limitations mentioned above, and additional double-blinded, randomized, placebo-controlled trials are also requested.

## Additional file

**Additional file 1: File S1.** Protocol amendments. **File S2.** Search strategy. **Table S1.** Study characteristics. **Table S2.** Risk of bias. **Table S3.** Meta-regression for primary outcome. **Table S4.** SUCRA values after adjusting for primary outcome. **Table S5.** Meta-regression for SBM. **Table S6.** GRADE Assessment for primary outcome. **Table S7.** GRADE Assessment for spontaneous bowel movements. **Table S8.** GRADE Assessment for Bristol score.

**Additional file 2:** Page S1. Node-splitting analysis for primary outcome. Page S2. Node-splitting analysis for spontaneous bowl movements. Page S3. Forest plot of median ranks for summarized three outcomes.

## Data Availability

Not applicable.

## Abbreviations

ACE: Acupoint catgut-embedding
AEs: Adverse effects
BS: Bristol score
CHD: Chinese herbal decoction
CIC: Chronic idiopathic constipation
CNKI: China National Knowledge Infrastructure
CSBS: Compound sodium bicarbonate suppository
CSCD: Chinese Science Citation Database
EA: Electroacupuncture
HS: Hemp Seed Pill
IF: Insoluble fiber
ITT: Intention-to-treat
LT: Lactulose
MCMC: Markov chain Monte Carlo
MD: Mean difference
MP: Mosapride
PB: Probiotics
PEG: Polyethylene glycol
PKU: Chinese Core Journals by Peking University
PP: Prucalopride
RCTs: Randomized controlled trials
RR: Risk ratio
RT: Recreation Therapy
SBMs: Spontaneous bowel movements
SF: Soluble fiber
SL: Stimulant laxatives
SUCRA: The Surface Under the Cumulative Ranking
TCM: Traditional Chinese Medicine
