# Supplementary material for: Comparative efficacy of pharmacological and nonpharmacological treatments for chronic idiopathic constipation in China: a Bayesian network meta-analysis
Source: BMC Complement Altern Med. 2019 Nov 14;19:311. doi: 10.1186/s12906-019-2741-z (PMC6857160; doi:10.1186/s12906-019-2741-z)
Supplement: Supplementary file 1 — Additional file 1: File S1. Protocol amendments. File S2. Search strategy. Table S1. Study characteristics. Table S2. Risk of bias. Table S3. Meta-regression for primary outcome. Table S4. SUCRA values after adjusting for primary outcome. Table S5. Meta-regression for SBM. Table S6. GRADE Assessment for primary outcome. Table S7. GRADE Assessment for spontaneous bowel movements. Table S8. GRADE Assessment for Bristol score. [file 12906_2019_2741_MOESM1_ESM.docx]

**S1 Appendix**

[File 1 Protocol amendments 2](#_Toc15664720)

[File 2 Search strategy 3](#_Toc15664721)

[Table 1 Study characteristics 6](#_Toc15664722)

[Table 2 Risk of bias 10](#_Toc15664723)

[Table 3 Meta-regression for primary outcome 13](#_Toc15664724)

[Table 4 SUCRA after adjustment for primary outcome 14](#_Toc15664725)

[Table 5 Meta-regression for SBM 15](#_Toc15664726)

[Table 6 GRADE Assessment for primary outcome 16](#_Toc15664727)

[Table 7 GRADE Assessment for spontaneous bowel movements 28](#_Toc15664728)

[Table 8 GRADE Assessment for Bristol score 39](#_Toc15664729)

# File 1 Protocol amendments

1. Because most studies reported the primary outcome as responders of spontaneous bowel movements (SBM) in treatment end point. Thus, we adjusted the primary outcome on SBM instead of Complete spontaneous bowel movements (CSBM) in all studies. It has been changed into proportion of responders based on ≥3 SBM/week.

2. We planned to use OpenBUGS as Markov Chain Monte Carlo engine to synthesize data. However, R package gemtc only support JAGS after updated.

# File 2 Search strategy

**PubMed**

#1 Search ((constipation[MeSH Terms]) OR chronic idiopathic constipation[Title/Abstract]) OR functional constipation[Title/Abstract]

13716

#2 Search (((((((randomized controlled study[Title/Abstract]) OR randomized controlled trial[Title/Abstract]) OR randomized study[Title/Abstract]) OR randomized trial[Title/Abstract]) OR randomized parallel-group study[Title/Abstract]) OR double-blinded controlled study[Title/Abstract]) OR randomized vehicle-controlled study[Title/Abstract]) OR randomized placebo-controlled study[Title/Abstract]

128714

#3 Search ((Meta-analysis[Title/Abstract]) OR systematic review[Title/Abstract]) OR protocol[Title/Abstract]

484587

#4 Search ((((children[Title/Abstract]) OR child[Title/Abstract]) OR teenager[Title/Abstract]) OR teens[Title/Abstract]) OR adolescent[Title/Abstract]

1263663

#5 Search ((((((constipation[MeSH Terms]) OR chronic idiopathic constipation[Title/Abstract]) OR functional constipation[Title/Abstract])) AND ((((((((randomized controlled study[Title/Abstract]) OR randomized controlled trial[Title/Abstract]) OR randomized study[Title/Abstract]) OR randomized trial[Title/Abstract]) OR randomized parallel-group study[Title/Abstract]) OR double-blinded controlled study[Title/Abstract]) OR randomized vehicle-controlled study[Title/Abstract]) OR randomized placebo-controlled study[Title/Abstract])) NOT (((Meta-analysis[Title/Abstract]) OR systematic review[Title/Abstract]) OR protocol[Title/Abstract])) NOT (((((children[Title/Abstract]) OR child[Title/Abstract]) OR teenager[Title/Abstract]) OR teens[Title/Abstract]) OR adolescent[Title/Abstract])

154

**Embase**

#1. 'chronic idiopathic constipation':ti,ab,kw OR 2,481

'functional constipation':ti,ab,kw

#2. 'randomized controlled study':ti,ab,kw OR 189,509

'randomized controlled trial':ti,ab,kw OR

'randomized study':ti,ab,kw OR 'randomized

trial':ti,ab,kw OR 'randomized parallel-group

study':ti,ab,kw OR 'double-blinded controlled

study':ti,ab,kw OR 'randomized vehicle-controlled

study':ti,ab,kw OR 'randomized placebo-controlled

study':ti,ab,kw

#3. 'meta analysis':ti,ab,kw OR 'systematic 685,866

review':ti,ab,kw OR protocol:ti,ab,kw

#4. children:ti,ab,kw OR child:ti,ab,kw OR 1,758,015

teenager:ti,ab,kw OR teens:ti,ab,kw OR

adolescent:ti,ab,kw

#5. #1 AND #2 NOT #3 NOT #4 35

**Cochrane library**

#1 (chronic idiopathic constipation):ti,ab,kw OR (functional constipation):ti,ab,kw

1414

#2 (randomized controlled study):ti,ab,kw OR (randomized controlled trial):ti,ab,kw OR (randomized study):ti,ab,kw OR (randomized parallel-group study):ti,ab,kw OR (double-blinded controlled study):ti,ab,kw

704483

#3 (Meta-analysis):ti,ab,kw OR (systematic review):ti,ab,kw OR (protocol):ti,ab,kw

107556

#4 (Meta-analysis):ti,ab,kw OR (systematic review):ti,ab,kw OR (protocol):ti,ab,kw

213520

#5 #1 AND #2 NOT #3 NOT #4

619

# Table 1 Study characteristics

| Study ID | Age (T/C) | Female (%) | Intervention | Control | Responders  (T/C) | Enrolment (T/C) | Length (weeks) | Outcomes |
| --- | --- | --- | --- | --- | --- | --- | --- | --- |
| Chen 2011 | 36.72/37.53 | 93.3% | Hemp Seed Pill | Placebo | 26/5 | 60/60 | 8 | ①②④ |
| Da 2015 | 37.94/37 | 80.6% | Electroacupuncture | Sham EA | 8/1 | 34/33 | 8 | ①②③ |
| Huang 2012 | 73.4/73.6 | 54.8% | Chinese herbal Decoction | Lactulose | NA | 44/49 | 8 | ②③④ |
| Ke 2012 | 41.4/41.8 | 89.8% | Prucalopride | Placebo | 83/26 | 249/252 | 12 | ①②④ |
| Liu 2016 | 47.01/47.33 | 76.5% | Electroacupuncture | Sham EA | 202/76 | 536/539 | 8 | ①②③④ |
| Wang 2005 | 51.23/50 | 59.5% | Polyethylene glycol | Psyllium Hydrophilic Mucilloid | 50/26 | 63/63 | 2 | ①②④ |
| Wu 2014a | 45.88/46.25/44.12 | 78.3% | Electroacupuncture | Sham EA/ Lactulose | NA | 237/119/119 | 8 | ②④ |
| Wu 2017 | 35.6/43.6 | 84.1% | Electroacupuncture | Mosapride | 74/35 | 130/71 | 4 | ①②③ |
| Xiong 2014 | 30.39/30.77 | 90.1% | Electroacupuncture | Mosapride | NA | 73/38 | 4 | ② |
| Xue 2015 | 48.85/45.25 | 83.3% | Electroacupuncture | Sham EA | 25/12 | 48/48 | 4 | ①④ |
| Yang 2008 | 46.4/46.4 | 100% | Probiotics | Placebo | NA | 63/63 | 2 | ② |
| Zheng 2018 | 39.8/40.6 | 81.2% | Electroacupuncture | Mosapride | NA | 505/170 | 4 | ②③④ |
| Zhong 2018 | 45.5/45.1/45.7 | 90% | Hemp Seed Pill (MaZiRenWan) | Senna/  Placebo | 66/56/32 | 97/97/97 | 8 | ①②④ |
| Cao 2012 | Na | Na | Probiotics+Lactulose | Lactulose | 26/13 | 32/32 | 2 | ①②④ |
| Chen 2017 | 44/39 | 100% | Acupoint catgut-embedding | Lactulose | NA | 22/26 | 8 | ②③ |
| Dai 2004 | 70.8 | 41.7% | Polyethylene glycol | Lactulose | 20/19 | 36/36 | 2 | ①②④ |
| Dai 2010 | NA | 47.5% | Stimulant laxatives | Lactulose | 28/20 | 38/39 | 2 | ①②④ |
| Fang 2002 | Na | Na | Polyethylene glycol | Lactulose | 61/44 | 92/94 | 4 | ①③④ |
| Fang 2017 | 69.9/68.9 | 52.5% | Dietary Fiber | Polyethylene glycol | 28/25 | 62/60 | 4 | ①④ |
| Guo 2018 | 46.65 | 56% | Prucalopride | Lactulose + Mosapride | 15/11 | 50/50 | 4 | ①④ |
| Huo 2016 | 72.6/72.4 | 46% | Mosapride + Chinese herbal decoction | Mosapride | 33/29 | 63/61 | 4 | ① |
| Jiao 2015 | 69.4/66.8 | 31% | Mosapride + Chinese herbal decoction | Mosapride | 11/7 | 50/50 | 3 | ①③ |
| Li 2015a | 41.14/41.5 | 64.6% | Probiotics+Lactulose | Lactulose | NA | 28/32 | 4 | ②③④ |
| Li 2012 | 46/45 | 75% | Probiotics+Lactulose | Lactulose | 44/37 | 104/112 | 4 | ①②③④ |
| Li 2015b | 46.1/44.57 | 62% | Chinese herbal decoction | Mosapride | 36/35 | 42/42 | 2 | ①②③ |
| Lian 2014 | 66.9/67.1 | 56% | Probiotics + Mosapride | Mosapride | 22/17 | 36/36 | 6 | ①④ |
| Luo 2012 | 72.4/71.5 | 57% | Probiotics + Mosapride | Stimulant laxatives | 27/12 | 34/34 | 4 | ① |
| Luo 2011 | 42.1/45 | 49.5% | Probiotics + Mosapride | Mosapride | 59/48 | 62/59 | 8 | ① |
| Lv 2017 | 47.6/51.4 | 76.1% | Electroacupuncture | Sham EA | 31/10 | 45/43 | 8 | ①③ |
| Peng 2013 | 52.5/59 | 66% | Electroacupuncture | Lactulose | 54/13 | 94/29 | 4 | ①②④ |
| Qian 2014 | 44.51/43.85/42.95 | 58.3% | Chinese herbal Decoction | Hemp Seed Pill/ Mosapride | 33/21/26 | 38/36/36 | 8 | ①④ |
| Ren 2005 | 40/30 | 71.7% | Mosapride | Placebo | NA | 30/30 | 2 | ②③ |
| Sun 2008 | Na | Na | Sodium Bicarbonate Suppository | Placebo | NA | 109/109 | 1 | ②③ |
| Wang 2010 | 48.8/40.8/44.6 | 84.2% | Electroacupuncture | Sham EA/ Lactulose | 37/14/4 | 48/24/23 | 4 | ①② |
| Wu 2014b | 49/52.63 | 85% | Electroacupuncture | Sham EA | 19/14 | 60/60 | 8 | ①②③ |
| Wu 2014c | 56.67/55 | 74% | Electroacupuncture | Mosapride | NA | 79/25 | 4 | ②④ |
| Xu 2014 | 45.3/42.2 | 61.25% | Dietary Fiber | Placebo | 26/7 | 40/40 | 4 | ① |
| Xu 2018 | 70.5/71.3 | 45% | Recreation Therapy | No treatment | NA | 56/56 | 8 | ②③ |
| Zhang 2013 | 55/57 | 53.3% | Electroacupuncture | Cisapride | 12/5 | 30/30 | 4 | ① |
| Zhang 2015 | 72.72/71.63 | 48.75% | Chinese herbal Decoction | Mosapride | NA | 40/40 | 4 | ②③④ |
| Zhang 2019 | 70/69.7 | 56% | Insoluble fibre + Prucalopride | Insoluble fibre | 27/21 | 45/45 | 4 | ①④ |
| Zhou 2001 | 52.25/49.95 | 49.4% | Polyethylene glycol | Psyllium Hydrophilic Mucilloid | 51/37 | 64/60 | 2 | ①②④ |

Footnotes: ①Responder rate; ②Spontaneous Bowel Movements per week; ③Bristol score; ④Adverse effects

# Table 2 Risk of bias

| Study ID | Generation of randomisation sequence | Allocation concealment | Double blind | Multi-center trial or not | ITT or not | Lost to follow-up |
| --- | --- | --- | --- | --- | --- | --- |
| Cheng 2011 | Random Allocation Software | + | + | No | Yes | 1Rx; 1C |
| Da 2015 | Stochastic systems in computer | - | participants-blind | No | Yes | 1C |
| Huang 2012 | Computer-generated randomization schedule | + | + | Yes | Yes | 2Rx; 2C |
| Ke 2012 | Computer-generated randomization schedule | + | + | Yes | Yes | 3Rx; 2C |
| Liu 2016 | PROC PLAN in SAS | + | + | Yes | Yes | 3Rx; 14C |
| Wang 2005 | Unclear | - | - | Yes | Yes | Unclear |
| Wu 2014a | Central Randomization System | + | + | Yes | Yes | 32Rx; 16C1; 17C2 |
| Wu 2017 | R 2.0 software | + | assessor-blind | Yes | Yes | 3Rx; 2C |
| Xiong 2014 | Unclear | - | assessor-blind | No | No | 2Rx; 1C |
| Xue 2015 | Central randomization | - | - | No | No | None |
| Yang 2008 | Unclear | - | + | No | Yes | Unclear |
| Zheng 2018 | Central computer system | + | assessor-blind | Yes | Yes | 10Rx; 7C |
| Zhong 2018 | Random Allocation Software 2.0 software | + | + | Yes | Yes | 2Rx; 2C1; 3C2 |
| Cao 2012 | Random number table | - | - | No | No | Unclear |
| Chen 2017 | Excel software | + | assessor-blind | No | Yes | 1 |
| Dai 2004 | Unclear | - | - | No | No | Unclear |
| Dai 2010 | Unclear | - | - | No | No | Unclear |
| Fang 2002 | Random number table | - | - | Yes | Yes | 7Rx; 12C |
| Fang 2017 | Random number table | - | + | Yes | No | 2Rx; 3C |
| Guo 2018 | Random number table | - | - | No | No | Unclear |
| Huo 2016 | Random number table | - | - | No | No | Unclear |
| Jiao 2015 | Unclear | - | - | No | No | Unclear |
| Li 2012 | Unclear | + | + | Yes | Yes | 6Rx; 6C |
| Li 2015a | Random number table | - | + | No | No | Unclear |
| Li 2015b | Random number table | - | - | No | No | 6Rx; 6C |
| Lian 2014 | Random number table | - | - | No | No | Unclear |
| Luo 2012 | Unclear | - | - | No | No | Unclear |
| Luo 2011 | Unclear | - | - | No | No | Unclear |
| Lv 2017 | Central computer system | + | - | No | No | 2C |
| Peng 2013 | Central computer system | + | + | Yes | Yes | None |
| Qian 2014 | Random number table | - | - | No | No | Unclear |
| Ren 2005 | Unclear | - | + | Yes | No | 2Rx |
| Sun 2008 | Unclear | - | + | Yes | Yes | 1Rx; 4C |
| Wang 2010 | Central computer system | - | - | No | Yes | 2C1; 3C2 |
| Wu 2014b | Central computer system | + | + | No | Yes | None |
| Wu 2014c | Central computer system | + | + | No | Yes | 3Rx; 1C |
| Xu 2014 | Random number table | - | + | No | No | Unclear |
| Xu 2018 | SPSS 18.0 software | - | assessor-blind | No | No | None |
| Zhang 2013 | quasi-random sequences | - | - | No | No | None |
| Zhang 2015 | Random number table | - | - | No | No | Unclear |
| Zhang 2019 | Unclear | - | - | No | No | Unclear |
| Zhou 2001 | Unclear | - | - | Yes | No | 2Rx; 1C |

# Table 3 Meta-regression for primary outcome

| Covariate | Median coefficient | 95%CrI | I-square | DIC |
| --- | --- | --- | --- | --- |
| No regression | NA | NA | 10% | 132 |
| Duration of treatment | -0.2479 | [-1.125, 0.698] | 10% | 132.7 |
| Course of disease | 0.5153 | [-0.184, 1.27] | 9% | 131 |
| Proportion of women | -0.554 | [-1.74, 0.58] | 9% | 131.6 |
| Sample size | -0.0677 | [-0.397, 0.23] | 9% | 131.8 |
| **Age** | **-2.5577** | **[-4.56, -0.697]** | **9%** | **128.4** |
| ITT or not | -0.17 | [-0.807, 0.506] | 10% | 132.5 |
| Low quality or not | 0.07 | [-9.627, 14.019] | 10% | 131.9 |
| Generation of randomization sequence or not | 0.0406 | [-15.436, 14.71] | 10% | 132 |
| Allocation concealment or not | -0.358 | [-1.063, 0.336] | 10% | 132.5 |
| Double blind or not | 0.035 | [-0.667, 0.69] | 9% | 132.4 |
| Multi-center trial or not | -0.1955 | [-0.819, 0.408] | 9% | 132 |
| **Lost to follow-up or not (>5%)** | **-1.0125** | **[-2.069, -0.0448]** | **11%** | **131** |

# Table 4 SUCRA after adjustment for primary outcome

| **SUCRA** | Original model | Power-adjust model | Uniform prior | Empirical prior | Half-normal prior | Adjusting for lost to follow-up | Adjusting for age |
| --- | --- | --- | --- | --- | --- | --- | --- |
| **PB+MP** | 0.79 | **0.67** | 0.79 | 0.8 | 0.78 | 0.83 | 0.85 |
| IF+PP | 0.72 | 0.64 | 0.72 | 0.73 | 0.71 | **0.62** | 0.76 |
| CHD | 0.7 | 0.66 | 0.7 | 0.7 | 0.69 | 0.75 | 0.73 |
| **PP** | 0.7 | **0.75** | 0.7 | 0.7 | 0.68 | 0.79 | **0.35** |
| EA | 0.68 | 0.66 | 0.68 | 0.68 | 0.67 | 0.72 | **0.82** |
| MP+CHD | 0.68 | 0.48 | 0.68 | 0.68 | 0.67 | 0.73 | 0.75 |
| **IF** | 0.58 | **0.69** | 0.58 | 0.58 | 0.58 | **0.45** | 0.59 |
| MP | 0.54 | 0.51 | 0.54 | 0.54 | 0.54 | 0.6 | 0.61 |
| LT+MP | 0.5 | 0.59 | 0.5 | 0.5 | 0.5 | 0.59 | **0.22** |
| PEG | 0.49 | 0.61 | 0.49 | 0.49 | 0.5 | **0.35** | 0.49 |
| LT+PB | 0.48 | 0.46 | 0.48 | 0.48 | 0.49 | 0.45 | 0.53 |
| HS | 0.46 | 0.51 | 0.46 | 0.46 | 0.48 | 0.51 | **0.37** |
| SL | 0.26 | 0.28 | 0.26 | 0.26 | 0.27 | 0.32 | 0.24 |
| SF | 0.25 | 0.27 | 0.25 | 0.24 | 0.27 | **0.12** | 0.17 |
| LT | 0.15 | 0.21 | 0.15 | 0.15 | 0.16 | 0.16 | 0.15 |
| Placebo | 0.01 | 0.02 | 0.01 | 0.01 | 0.01 | 0 | 0.38 |

# Table 5 Meta-regression for SBM

| Covariate | Median coefficient | 95%CrI | I-square | DIC |
| --- | --- | --- | --- | --- |
| No regression | NA | NA | 2% | 131.2 |
| Duration of treatment | -1.099 | [-2.63, 0.69] | 3% | 131.3 |
| Course of disease | -1.53 | [-3.17, 0.187] | 3% | 131 |
| Proportion of women | -2.223 | [-6.05, 0.956] | 3% | 131.4 |
| Sample size | -0.044 | [-0.95, 0.79] | 2% | 131.4 |
| Age | -0.77 | [-3.38, 1.8] | 2% | 131.4 |
| ITT or not | -1.15 | [-2.89, 0.617] | 3% | 131.5 |
| Low quality or not | -0.136 | [-26.7, 20.9] | 2% | 131 |
| Generation of randomization sequence or not | -1.158 | [-2.97, 0.633] | 3% | 131.5 |
| Allocation concealment or not | -0.78 | [-2.3, 0.71] | 2% | 131.4 |
| Double blind or not | 0.016 | [-1.88, 1.96] | 2% | 131.6 |
| Multi-center trial or not | -0.11 | [-1.46, 1.167] | 2% | 131.4 |
| Lost to follow-up or not (>5%) | 0.667 | [-1.05, 2.4] | 2% | 131 |

# Table 6 GRADE Assessment for primary outcome

| **Comparison** | **Direct evidence** | | **Indirect evidence** | | **Network meta-analysis** | |
| --- | --- | --- | --- | --- | --- | --- |
|  | **Risk ratio**  **(95%CrI)** | **Quality of**  **evidence** | **Risk ratio**  **(95%CrI)** | **Quality of**  **evidence** | **Risk ratio**  **(95%CrI)** | **Quality of**  **evidence** |
| HS vs CHD | 0.66 (0.30, 1.4) | Low^1,3^ | 1.1 (0.40, 3.4) | Low | 0.78 (0.46, 1.4) | Low^4^ |
| LT vs EA | 0.50 (0.31, 0.77) | Moderate^2^ | 0.68 (0.32, 1.5) | Low | 0.55 (0.37, 0.78) | Moderate |
| MP vs EA | 0.78 (0.49, 1.1) | Low^1,3^ | 1.4 (0.69, 2.9) | Low | 0.89 (0.61, 1.3) | Low |
| Placebo vs EA | 0.41 (0.28, 0.57) | High | 0.21 (0.11, 0.36) | Low | 0.34 (0.24, 0.46) | Moderate^7^ |
| MP vs HS | 1.2 (0.56, 2.7) | Low^1,3^ | 0.89 (0.39, 1.8) | Low | 1.1 (0.67, 1.7) | Low |
| Placebo vs HS | 0.36 (0.18, 0.63) | Moderate^2^ | 0.57 (0.23, 1.5) | High | 0.42 (0.26, 0.64) | High |
| SL vs HS | 0.84 (0.40, 1.8) | High | 0.70 (0.26, 1.7) | Moderate | 0.80 (0.47, 1.3) | High |
| PEG vs LT | 1.3 (0.83, 1.9) | Moderate^1^ | 4.0 (1.5, 12.) | Low | 1.5 (0.98, 2.4) | Very low^4,7^ |
| SL vs LT | 1.4 (0.69, 2.9) | Moderate^1^ | 0.98 (0.50, 2.0) | Low | 1.2 (0.73, 1.9) | Moderate |
| PB+MP vs MP | 1.2 (0.74, 2.0) | Moderate^1^ | 1.9 (0.72, 5.4) | Low | 1.3 (0.87, 2.0) | Moderate |
| SL vs PB+MP | 0.43 (0.19, 0.94) | Low^1,3^ | 0.69 (0.32, 1.5) | Low | 0.56 (0.32, 0.93) | Low |
| SF vs PEG | 0.64 (0.43, 0.95) | Low^1,2^ | 2.0 (0.76, 6.) | Low | 0.75 (0.50, 1.2) | Very low^4,7^ |
| SF vs Placebo | 3.4 (1.6, 8.4) | Moderate^3^ | 1.1 (0.52, 2.2) | Low | 1.8 (1.0, 3.4) | Very low^4,7^ |
| SL vs Placebo | 1.7 (0.78, 3.8) | High | 1.9 (0.89, 4.3) | Moderate | 1.9 (1.2, 3.1) | High |
| MP vs CHD | 0.90 (0.58, 1.4) | Low^1,3^ | — | — | 0.86 (0.54, 1.3) | Low |
| IF+PP vs IF | 1.3 (0.64, 2.5) | Moderate^1^ | — | — | 1.3 (0.61, 2.6) | Moderate |
| PEG vs IF | 0.89 (0.45, 1.8) | High | — | — | 0.89 (0.43, 1.8) | High |
| LT+PB vs LT | 1.5 (0.92, 2.4) | High | — | — | 1.5 (0.92, 2.5) | High |
| PP vs LT+MP | 1.3 (0.55, 3.1) | High | — | — | 1.3 (0.52, 3.2) | High |
| MP+CHD vs MP | 1.2 (0.69, 2.0) | Low^1,3^ | — | — | 1.2 (0.67, 2.1) | Low |
| PP vs Placebo | 3.2 (1.6, 6.2) | High | — | — | 3.2 (1.5, 6.6) | High |
| CHD vs EA | — | — | 1.04  (0.6, 1.79) | Low | 1.04  (0.6, 1.79) | Low |
| CHD vs IF | — | — | 1.28  (0.72, 2.18) | Very low^6^ | 1.28  (0.72, 2.18) | Very low |
| CHD vs IF+PP | — | — | 1.12  (0.4, 3.09) | Very low^6^ | 1.12  (0.4, 3.09) | Very low |
| CHD vs LT | — | — | 0.89  (0.25, 3.04) | Low | 0.89  (0.25, 3.04) | Low |
| CHD vs LT+MP | — | — | 1.89  (1.02, 3.61) | Very low^6^ | 1.89  (1.02, 3.61) | Very low |
| CHD vs LT+PB | — | — | 1.23  (0.34, 4.6) | Very low^6^ | 1.23  (0.34, 4.6) | Very low |
| CHD vs MP+CHD | — | — | 1.27  (0.57, 2.85) | Low | 1.27  (0.57, 2.85) | Low |
| CHD vs PB+MP | — | — | 1.16  (0.76, 1.84) | Low | 1.16  (0.76, 1.84) | Low |
| CHD vs PEG | — | — | 0.99  (0.48, 2.03) | Low | 0.99  (0.48, 2.03) | Low |
| CHD vs Placebo | — | — | 0.9  (0.49, 1.63) | Low | 0.9  (0.49, 1.63) | Low |
| CHD vs PP | — | — | 1.26  (0.6, 2.6) | Low | 1.26  (0.6, 2.6) | Low |
| CHD vs SF | — | — | 3.05  (1.72, 5.54) | Low | 3.05  (1.72, 5.54) | Low |
| CHD vs SL | — | — | 0.96  (0.38, 2.49) | Low | 0.96  (0.38, 2.49) | Low |
| EA vs HS | — | — | 1.23  (0.75, 1.94) | Low | 1.23  (0.75, 1.94) | Low |
| EA vs IF | — | — | 1.08  (0.44, 2.63) | Moderate | 1.08  (0.44, 2.63) | Moderate |
| EA vs IF+PP | — | — | 0.86  (0.27, 2.68) | Moderate | 0.86  (0.27, 2.68) | Moderate |
| EA vs LT+MP | — | — | 1.82  (1.27, 2.65) | Moderate^6^ | 1.82  (1.27, 2.65) | Moderate |
| EA vs LT+PB | — | — | 1.19  (0.37, 3.97) | Moderate | 1.19  (0.37, 3.97) | Moderate |
| EA vs MP+CHD | — | — | 1.22  (0.66, 2.28) | Low | 1.22  (0.66, 2.28) | Low |
| EA vs PB+MP | — | — | 1.12  (0.79, 1.65) | Low | 1.12  (0.79, 1.65) | Low |
| EA vs PEG | — | — | 0.96  (0.48, 1.9) | Moderate | 0.96  (0.48, 1.9) | Moderate |
| EA vs PP | — | — | 0.86  (0.51, 1.45) | High | 0.86  (0.51, 1.45) | High |
| EA vs SF | — | — | 1.21  (0.71, 2.07) | Low | 1.21  (0.71, 2.07) | Low |
| EA vs SL | — | — | 2.93  (2.15, 4.14) | Moderate | 2.93  (2.15, 4.14) | Moderate |
| HS vs IF | — | — | 0.88  (0.34, 2.38) | Very low^6^ | 0.88  (0.34, 2.38) | Very low |
| HS vs IF+PP | — | — | 0.7  (0.21, 2.37) | Very low^6^ | 0.7  (0.21, 2.37) | Very low |
| HS vs LT | — | — | 1.49  (0.87, 2.66) | Low | 1.49  (0.87, 2.66) | Low |
| HS vs LT+MP | — | — | 0.97  (0.29, 3.39) | Moderate | 0.97  (0.29, 3.39) | Moderate |
| HS vs LT+PB | — | — | 1  (0.48, 2.13) | Moderate | 1  (0.48, 2.13) | Moderate |
| HS vs MP+CHD | — | — | 0.91  (0.59, 1.5) | Low | 0.91  (0.59, 1.5) | Low |
| HS vs PB+MP | — | — | 0.78  (0.37, 1.66) | Low | 0.78  (0.37, 1.66) | Low |
| HS vs PEG | — | — | 0.7  (0.4, 1.28) | Moderate | 0.7  (0.4, 1.28) | Moderate |
| HS vs PP | — | — | 0.99  (0.52, 1.94) | Moderate | 0.99  (0.52, 1.94) | Moderate |
| HS vs SF | — | — | 2.39  (1.56, 3.89) | Moderate | 2.39  (1.56, 3.89) | Moderate |
| IF vs LT | — | — | 1.69  (0.73, 3.97) | Moderate | 1.69  (0.73, 3.97) | Moderate |
| IF vs LT+MP | — | — | 1.11  (0.25, 4.85) | Very low^6^ | 1.11  (0.25, 4.85) | Very low |
| IF vs LT+PB | — | — | 1.13  (0.43, 3.05) | Moderate | 1.13  (0.43, 3.05) | Moderate |
| IF vs MP | — | — | 1.03  (0.41, 2.7) | Low | 1.03  (0.41, 2.7) | Low |
| IF vs MP+CHD | — | — | 0.88  (0.29, 2.7) | Low | 0.88  (0.29, 2.7) | Low |
| IF vs PB+MP | — | — | 0.8  (0.29, 2.16) | Low | 0.8  (0.29, 2.16) | Low |
| IF vs Placebo | — | — | 1.12  (0.54, 2.34) | Low | 1.12  (0.54, 2.34) | Low |
| IF vs PP | — | — | 2.7  (1.1, 6.84) | Low | 2.7  (1.1, 6.84) | Low |
| IF vs SF | — | — | 0.86  (0.27, 2.85) | Low | 0.86  (0.27, 2.85) | Low |
| IF vs SL | — | — | 1.5  (0.63, 3.42) | Moderate | 1.5  (0.63, 3.42) | Moderate |
| IF+PP vs LT | — | — | 2.13  (0.72, 6.62) | Moderate | 2.13  (0.72, 6.62) | Moderate |
| IF+PP vs LT+MP | — | — | 1.38  (0.27, 7.3) | Very low^6^ | 1.38  (0.27, 7.3) | Very low |
| IF+PP vs LT+PB | — | — | 1.42  (0.43, 4.91) | Moderate | 1.42  (0.43, 4.91) | Moderate |
| IF+PP vs MP | — | — | 1.29  (0.41, 4.36) | Very low^6^ | 1.29  (0.41, 4.36) | Very low |
| IF+PP vs MP+CHD | — | — | 1.11  (0.3, 4.23) | Very low^6^ | 1.11  (0.3, 4.23) | Very low |
| IF+PP vs PB+MP | — | — | 1  (0.29, 3.42) | Low | 1  (0.29, 3.42) | Low |
| IF+PP vs PEG | — | — | 1.4  (0.51, 3.99) | Moderate | 1.4  (0.51, 3.99) | Moderate |
| IF+PP vs Placebo | — | — | 3.41  (1.1, 11.06) | Low | 3.41  (1.1, 11.06) | Low |
| IF+PP vs PP | — | — | 1.08  (0.28, 4.3) | Low | 1.08  (0.28, 4.3) | Low |
| IF+PP vs SF | — | — | 1.88  (0.61, 5.7) | Low | 1.88  (0.61, 5.7) | Low |
| IF+PP vs SL | — | — | 1.8  (0.54, 6.03) | Moderate | 1.8  (0.54, 6.03) | Moderate |
| LT vs LT+MP | — | — | 0.65  (0.19, 2.23) | Very low^6^ | 0.65  (0.19, 2.23) | Very low |
| LT vs MP | — | — | 0.67  (0.4, 1.1) | Low | 0.67  (0.4, 1.1) | Low |
| LT vs MP+CHD | — | — | 0.61  (0.38, 1.01) | Low | 0.61  (0.38, 1.01) | Low |
| LT vs PB+MP | — | — | 0.53  (0.24, 1.11) | Low | 0.53  (0.24, 1.11) | Low |
| LT vs Placebo | — | — | 0.48  (0.26, 0.84) | Moderate | 0.48  (0.26, 0.84) | Moderate |
| LT vs PP | — | — | 0.67  (0.42, 1.02) | Moderate | 0.67  (0.42, 1.02) | Moderate |
| LT vs SF | — | — | 1.61  (1.05, 2.51) | Low | 1.61  (1.05, 2.51) | Low |
| LT+MP vs LT+PB | — | — | 1.03  (0.28, 3.85) | Low^6^ | 1.03  (0.28, 3.85) | Low |
| LT+MP vs MP | — | — | 0.94  (0.28, 3.26) | Low | 0.94  (0.28, 3.26) | Low |
| LT+MP vs MP+CHD | — | — | 0.8  (0.2, 3.16) | Very low^6^ | 0.8  (0.2, 3.16) | Very low |
| LT+MP vs PB+MP | — | — | 0.73  (0.2, 2.6) | Low | 0.73  (0.2, 2.6) | Low |
| LT+MP vs PEG | — | — | 1.02  (0.28, 3.61) | Very low^6^ | 1.02  (0.28, 3.61) | Very low |
| LT+MP vs Placebo | — | — | 2.47  (0.79, 7.87) | Moderate^6^ | 2.47  (0.79, 7.87) | Moderate |
| LT+MP vs SF | — | — | 0.78  (0.32, 1.91) | Low^6^ | 0.78  (0.32, 1.91) | Low |
| LT+MP vs SL | — | — | 1.35  (0.36, 4.83) | Moderate^6^ | 1.35  (0.36, 4.83) | Moderate |
| LT+PB vs MP | — | — | 0.92  (0.46, 1.86) | Low | 0.92  (0.46, 1.86) | Low |
| LT+PB vs MP+CHD | — | — | 0.78  (0.31, 1.92) | Low | 0.78  (0.31, 1.92) | Low |
| LT+PB vs PB+MP | — | — | 0.71  (0.32, 1.53) | Low | 0.71  (0.32, 1.53) | Low |
| LT+PB vs PEG | — | — | 0.99  (0.51, 1.91) | Moderate | 0.99  (0.51, 1.91) | Moderate |
| LT+PB vs Placebo | — | — | 2.4  (1.25, 4.74) | Moderate | 2.4  (1.25, 4.74) | Moderate |
| LT+PB vs PP | — | — | 0.76  (0.29, 2.03) | Moderate | 0.76  (0.29, 2.03) | Moderate |
| LT+PB vs SF | — | — | 1.33  (0.6, 2.75) | Moderate | 1.33  (0.6, 2.75) | Moderate |
| LT+PB vs SL | — | — | 1.27  (0.62, 2.57) | Moderate | 1.27  (0.62, 2.57) | Moderate |
| MP vs PEG | — | — | 1.08  (0.57, 1.99) | Low | 1.08  (0.57, 1.99) | Low |
| MP vs Placebo | — | — | 2.63  (1.69, 4.07) | Low | 2.63  (1.69, 4.07) | Low |
| MP vs PP | — | — | 0.83  (0.35, 1.93) | Low | 0.83  (0.35, 1.93) | Low |
| MP vs SF | — | — | 1.45  (0.72, 2.73) | Low | 1.45  (0.72, 2.73) | Low |
| MP vs SL | — | — | 1.38  (0.84, 2.27) | Low | 1.38  (0.84, 2.27) | Low |
| MP+CHD vs PB+MP | — | — | 0.9  (0.45, 1.83) | Low | 0.9  (0.45, 1.83) | Low |
| MP+CHD vs PEG | — | — | 1.27  (0.54, 2.97) | Low | 1.27  (0.54, 2.97) | Low |
| MP+CHD vs Placebo | — | — | 3.07  (1.52, 6.43) | Low | 3.07  (1.52, 6.43) | Low |
| MP+CHD vs PP | — | — | 0.97  (0.35, 2.73) | Low | 0.97  (0.35, 2.73) | Low |
| MP+CHD vs SF | — | — | 1.69  (0.68, 4.04) | Low | 1.69  (0.68, 4.04) | Low |
| MP+CHD vs SL | — | — | 1.62  (0.77, 3.51) | Low | 1.62  (0.77, 3.51) | Low |
| PB+MP vs PEG | — | — | 1.4  (0.71, 2.86) | Low | 1.4  (0.71, 2.86) | Low |
| PB+MP vs Placebo | — | — | 3.4  (1.98, 6.14) | Low | 3.4  (1.98, 6.14) | Low |
| PB+MP vs PP | — | — | 1.08  (0.44, 2.73) | Low | 1.08  (0.44, 2.73) | Low |
| PB+MP vs SF | — | — | 1.88  (0.87, 3.9) | Low | 1.88  (0.87, 3.9) | Low |
| PEG vs Placebo | — | — | 2.42  (1.42, 4.3) | Low | 2.42  (1.42, 4.3) | Low |
| PEG vs PP | — | — | 0.76  (0.31, 1.94) | Low | 0.76  (0.31, 1.94) | Low |
| PEG vs SL | — | — | 1.34  (0.84, 2) | Low | 1.34  (0.84, 2) | Low |
| PP vs SF | — | — | 1.74  (0.66, 4.38) | Moderate | 1.74  (0.66, 4.38) | Moderate |
| PP vs SL | — | — | 1.67  (0.69, 4) | Moderate^6^ | 1.67  (0.69, 4) | Moderate |
| SF vs SL | — | — | 0.96  (0.5, 1.95) | Moderate | 0.96  (0.5, 1.95) | Moderate |

**GRADE Assessment:** Reasons for downgrading evidence (1 to 7): 1. Downgrading due to Risk of Bias; 2. Downgrading due to Inconsistency; 3. Downgrading due to Indirectness; 4. Downgrading due to Imprecision; 5. Downgrading due to Publication Bias; 6. Downgrading due to Intransitivity (for indirect evidence); 7. Downgrading due to Incoherence (for mixed estimates).

# Table 7 GRADE Assessment for spontaneous bowel movements

| **Comparison** | **Direct evidence** | | **Indirect evidence** | | **Network meta-analysis** | |
| --- | --- | --- | --- | --- | --- | --- |
|  | **Risk ratio**  **(95%CrI)** | **Quality of**  **evidence** | **Risk ratio**  **(95%CrI)** | **Quality of**  **evidence** | **Risk ratio**  **(95%CrI)** | **Quality of**  **evidence** |
| LT vs CHD | −1.2 (−3.3, 0.86) | High | −1.9 (−3.5, −0.11) | Low | −1.6 (−2.9, −0.28) | High |
| MP vs CHD | −1.7 (−2.9, −0.25) | Very low^1,2,5^ | −0.98 (−3.3, 1.3) | Moderate | −1.5 (−2.6, −0.29) | Moderate |
| LT vs EA | 0.13 (−0.67, 0.93) | Moderate^5^ | −0.73 (−2.5, 1.1) | Low | −0.012 (−0.73, 0.71) | Moderate |
| MP vs EA | −0.023 (−0.79, 0.75) | High | 0.66 (−0.86, 2.2) | Low | 0.12 (−0.57, 0.80) | High |
| Placebo vs EA | −0.60 (−1.5, 0.29) | Moderate^2^ | −0.65 (−2.2, 0.90) | Low | −0.62 (−1.4, 0.13) | Moderate |
| SL vs HS | −0.20 (−1.9, 1.5) | High | 1.6 (−1.0, 4.1) | High | 0.28 (−1.2, 1.7) | High |
| SL vs LT | 2.1 (0.51, 3.7) | Low^1,3^ | −0.057 (−1.9, 1.8) | Moderate | 1.2 (−0.11, 2.5) | Low^4^ |
| Placebo vs MP | −1.6 (−3.3, −0.035) | Low^3,5^ | −0.33 (−1.4, 0.72) | Moderate | −0.74 (−1.7, 0.18) | Moderate |
| SL vs Placebo | 0.80 (−0.87, 2.5) | High | 3.1 (1.1, 5.) | High | 1.8 (0.50, 3.0) | High |
| LT vs ACE | −0.38 (−2., 1.2) | Moderate^5^ | — | — | −0.39 (−2.1, 1.3) | Moderate |
| Placebo vs CSBS | −2.9 (−4.5, −1.3) | High | — | — | −2.9 (−4.6, −1.1) | High |
| Placebo vs HS | −1.3 (−2.4, −0.067) | High | — | — | −1.5 (−2.7, −0.29) | High |
| LT+PB vs LT | 1.1 (0.21, 2.2) | Low^2,5^ | — | — | 1.2 (0.15, 2.3) | Low |
| PEG vs LT | 0.28 (−1.3, 1.9) | Low^1,3^ | — | — | 0.29 (−1.4, 2.0) | Low |
| Placebo vs PB | −1.5 (−3.1, 0.087) | Low^1,3^ | — | — | −1.5 (−3.2, 0.21) | Low |
| SF vs PEG | −1.9 (−3.2, −0.75) | Very low^1,2,5^ | — | — | −1.9 (−3.3, −0.67) | Very low |
| PP vs Placebo | 1.3 (−0.27, 2.8) | High | — | — | 1.3 (−0.37, 3.) | High |
| RT vs Placebo | 2.4 (0.70, 4.1) | High | — | — | 2.4 (0.61, 4.2) | High |
| CHD vs ACE | — | — | 1.24  (-0.92, 3.33) | Low | 1.24  (-0.92, 3.33) | Low |
| CSBS vs ACE | — | — | 1.9  (-0.73, 4.56) | Low^6^ | 1.9  (-0.73, 4.56) | Low |
| CSBS vs CHD | — | — | 0.66  (-1.52, 2.92) | Moderate^6^ | 0.66  (-1.52, 2.92) | Moderate |
| EA vs ACE | — | — | -0.38  (-2.22, 1.52) | Moderate | -0.38  (-2.22, 1.52) | Moderate |
| EA vs CHD | — | — | -1.62  (-2.78, -0.34) | Very low | -1.62  (-2.78, -0.34) | Very low |
| EA vs CSBS | — | — | -2.27  (-4.15, -0.4) | Moderate | -2.27  (-4.15, -0.4) | Moderate |
| HS vs ACE | — | — | 0.5  (-1.73, 2.8) | Very low^6^ | 0.5  (-1.73, 2.8) | Very low |
| HS vs CHD | — | — | -0.73  (-2.45, 1.1) | Low^6^ | -0.73  (-2.45, 1.1) | Low |
| HS vs CSBS | — | — | -1.39  (-3.49, 0.71) | High | -1.39  (-3.49, 0.71) | High |
| HS vs EA | — | — | 0.89  (-0.47, 2.25) | Moderate | 0.89  (-0.47, 2.25) | Moderate |
| LT vs CSBS | — | — | -2.29  (-4.27, -0.31) | Moderate | -2.29  (-4.27, -0.31) | Moderate |
| LT vs HS | — | — | -0.01  (-0.73, 0.71) | Low | -0.01  (-0.73, 0.71) | Low |
| LT+PB vs ACE | — | — | 0.77  (-1.2, 2.82) | Low | 0.77  (-1.2, 2.82) | Low |
| LT+PB vs CHD | — | — | -0.47  (-2.03, 1.3) | Low | -0.47  (-2.03, 1.3) | Low |
| LT+PB vs CSBS | — | — | -1.12  (-3.35, 1.14) | Low^6^ | -1.12  (-3.35, 1.14) | Low |
| LT+PB vs EA | — | — | 1.15  (-0.04, 2.45) | Low | 1.15  (-0.04, 2.45) | Low |
| LT+PB vs HS | — | — | 0.27  (-1.46, 2.07) | Very low^6^ | 0.27  (-1.46, 2.07) | Very low |
| MP vs ACE | — | — | -0.27  (-2.2, 1.73) | Moderate | -0.27  (-2.2, 1.73) | Moderate |
| MP vs CSBS | — | — | -1.51  (-2.59, -0.31) | Moderate | -1.51  (-2.59, -0.31) | Moderate |
| MP vs HS | — | — | -2.16  (-4.11, -0.2) | Low | -2.16  (-4.11, -0.2) | Low |
| MP vs LT | — | — | 0.11  (-0.58, 0.79) | Moderate | 0.11  (-0.58, 0.79) | Moderate |
| MP vs LT+PB | — | — | -0.77  (-2.22, 0.67) | Low | -0.77  (-2.22, 0.67) | Low |
| PB vs ACE | — | — | 0.51  (-2.05, 3.16) | Very low^6^ | 0.51  (-2.05, 3.16) | Very low |
| PB vs CHD | — | — | -0.75  (-2.88, 1.52) | Low | -0.75  (-2.88, 1.52) | Low |
| PB vs CSBS | — | — | -1.4  (-3.85, 1) | Moderate | -1.4  (-3.85, 1) | Moderate |
| PB vs EA | — | — | 0.88  (-0.96, 2.76) | Low | 0.88  (-0.96, 2.76) | Low |
| PB vs HS | — | — | 0  (-2.07, 2.08) | Low | 0  (-2.07, 2.08) | Low |
| PB vs LT | — | — | 0.89  (-1.03, 2.88) | Low | 0.89  (-1.03, 2.88) | Low |
| PB vs LT+PB | — | — | -0.27  (-2.49, 1.91) | Very low^6^ | -0.27  (-2.49, 1.91) | Very low |
| PB vs MP | — | — | 0.76  (-1.17, 2.71) | Low | 0.76  (-1.17, 2.71) | Low |
| PEG vs ACE | — | — | -0.12  (-2.51, 2.32) | Low | -0.12  (-2.51, 2.32) | Low |
| PEG vs CHD | — | — | -1.35  (-3.47, 0.87) | Low | -1.35  (-3.47, 0.87) | Low |
| PEG vs CSBS | — | — | -2  (-4.58, 0.61) | Low^6^ | -2  (-4.58, 0.61) | Low |
| PEG vs EA | — | — | 0.27  (-1.61, 2.11) | Low | 0.27  (-1.61, 2.11) | Low |
| PEG vs HS | — | — | -0.62  (-2.86, 1.65) | Very low^6^ | -0.62  (-2.86, 1.65) | Very low |
| PEG vs LT+PB | — | — | 0.27  (-1.44, 2.01) | Low | 0.27  (-1.44, 2.01) | Low |
| PEG vs MP | — | — | -0.89  (-2.94, 1.06) | Low | -0.89  (-2.94, 1.06) | Low |
| PEG vs PB | — | — | 0.15  (-1.81, 2.13) | Very low^6^ | 0.15  (-1.81, 2.13) | Very low |
| Placebo vs ACE | — | — | -1  (-2.96, 1.02) | Moderate | -1  (-2.96, 1.02) | Moderate |
| Placebo vs CHD | — | — | -2.24  (-3.56, -0.81) | Very low | -2.24  (-3.56, -0.81) | Very low |
| Placebo vs LT | — | — | -2.9  (-4.62, -1.15) | Moderate | -2.9  (-4.62, -1.15) | Moderate |
| Placebo vs LT+PB | — | — | -0.62  (-1.36, 0.14) | Low | -0.62  (-1.36, 0.14) | Low |
| Placebo vs PEG | — | — | -1.5  (-2.7, -0.29) | Low | -1.5  (-2.7, -0.29) | Low |
| PP vs ACE | — | — | 0.3  (-2.23, 2.89) | Low^6^ | 0.3  (-2.23, 2.89) | Low |
| PP vs CHD | — | — | -0.95  (-3.06, 1.32) | Very low | -0.95  (-3.06, 1.32) | Very low |
| PP vs CSBS | — | — | -1.6  (-3.97, 0.83) | High | -1.6  (-3.97, 0.83) | High |
| PP vs EA | — | — | 0.67  (-1.16, 2.51) | Moderate | 0.67  (-1.16, 2.51) | Moderate |
| PP vs HS | — | — | -0.2  (-2.29, 1.82) | High | -0.2  (-2.29, 1.82) | High |
| PP vs LT | — | — | 0.69  (-1.24, 2.63) | Moderate | 0.69  (-1.24, 2.63) | Moderate |
| PP vs LT+PB | — | — | -0.47  (-2.7, 1.67) | Very low^6^ | -0.47  (-2.7, 1.67) | Very low |
| PP vs MP | — | — | 0.57  (-1.34, 2.47) | Low | 0.57  (-1.34, 2.47) | Low |
| PP vs PB | — | — | -0.21  (-2.57, 2.15) | Low | -0.21  (-2.57, 2.15) | Low |
| PP vs PEG | — | — | 0.41  (-2.18, 3.03) | Very low^6^ | 0.41  (-2.18, 3.03) | Very low |
| RT vs ACE | — | — | 1.41  (-1.28, 4.08) | Low^6^ | 1.41  (-1.28, 4.08) | Low |
| RT vs CHD | — | — | 0.18  (-2.06, 2.49) | Low^6^ | 0.18  (-2.06, 2.49) | Low |
| RT vs CSBS | — | — | -0.48  (-2.97, 2.02) | High | -0.48  (-2.97, 2.02) | High |
| RT vs EA | — | — | 1.8  (-0.18, 3.76) | Moderate | 1.8  (-0.18, 3.76) | Moderate |
| RT vs HS | — | — | 0.91  (-1.28, 3.07) | High | 0.91  (-1.28, 3.07) | High |
| RT vs LT | — | — | 1.81  (-0.24, 3.86) | Moderate | 1.81  (-0.24, 3.86) | Moderate |
| RT vs LT+PB | — | — | 0.64  (-1.67, 2.91) | Low^6^ | 0.64  (-1.67, 2.91) | Low |
| RT vs MP | — | — | 1.69  (-0.37, 3.7) | Moderate | 1.69  (-0.37, 3.7) | Moderate |
| RT vs PB | — | — | 0.9  (-1.57, 3.38) | Moderate | 0.9  (-1.57, 3.38) | Moderate |
| RT vs PEG | — | — | 1.52  (-1.15, 4.21) | Low^6^ | 1.52  (-1.15, 4.21) | Low |
| RT vs PP | — | — | 2.41  (0.61, 4.22) | High | 2.41  (0.61, 4.22) | High |
| SF vs ACE | — | — | -2.05  (-4.8, 0.66) | Low | -2.05  (-4.8, 0.66) | Low |
| SF vs CHD | — | — | -3.29  (-5.77, -0.77) | Low | -3.29  (-5.77, -0.77) | Low |
| SF vs CSBS | — | — | -3.95  (-6.84, -1.1) | Low^6^ | -3.95  (-6.84, -1.1) | Low |
| SF vs EA | — | — | -1.67  (-3.94, 0.58) | Low | -1.67  (-3.94, 0.58) | Low |
| SF vs HS | — | — | -2.57  (-5.17, 0.04) | Very low^6^ | -2.57  (-5.17, 0.04) | Very low |
| SF vs LT | — | — | -1.66  (-3.81, 0.47) | Very low | -1.66  (-3.81, 0.47) | Very low |
| SF vs LT+PB | — | — | -2.83  (-5.26, -0.49) | Very low | -2.83  (-5.26, -0.49) | Very low |
| SF vs MP | — | — | -1.79  (-4.15, 0.55) | Very low^6^ | -1.79  (-4.15, 0.55) | Very low |
| SF vs PB | — | — | -2.56  (-5.45, 0.29) | Very low^6^ | -2.56  (-5.45, 0.29) | Very low |
| SF vs Placebo | — | — | -1.94  (-3.27, -0.69) | Very low^6^ | -1.94  (-3.27, -0.69) | Very low |
| SF vs PP | — | — | -1.05  (-3.43, 1.28) | Very low^6^ | -1.05  (-3.43, 1.28) | Very low |
| SF vs RT | — | — | -2.35  (-5.3, 0.51) | Low^6^ | -2.35  (-5.3, 0.51) | Low |
| SL vs ACE | — | — | 0.79  (-1.35, 2.96) | Low | 0.79  (-1.35, 2.96) | Low |
| SL vs CHD | — | — | -0.45  (-2.11, 1.34) | Moderate | -0.45  (-2.11, 1.34) | Moderate |
| SL vs CSBS | — | — | -1.11  (-3.28, 1.05) | High | -1.11  (-3.28, 1.05) | High |
| SL vs EA | — | — | 1.17  (-0.13, 2.48) | Moderate | 1.17  (-0.13, 2.48) | Moderate |
| SL vs LT+PB | — | — | 0.29  (-1.17, 1.73) | Low | 0.29  (-1.17, 1.73) | Low |
| SL vs MP | — | — | 1.18  (-0.1, 2.48) | Moderate | 1.18  (-0.1, 2.48) | Moderate |
| SL vs PB | — | — | 0.02  (-1.69, 1.62) | Very low^6^ | 0.02  (-1.69, 1.62) | Very low |
| SL vs PEG | — | — | 1.06  (-0.38, 2.48) | Low | 1.06  (-0.38, 2.48) | Low |
| SL vs PP | — | — | 0.29  (-1.83, 2.43) | High | 0.29  (-1.83, 2.43) | High |
| SL vs RT | — | — | 0.9  (-1.28, 3.07) | High | 0.9  (-1.28, 3.07) | High |
| SL vs SF | — | — | 1.78  (0.51, 3.06) | Very low | 1.78  (0.51, 3.06) | Very low |

**GRADE Assessment:** Reasons for downgrading evidence (1 to 7): 1. Downgrading due to Risk of Bias; 2. Downgrading due to Inconsistency; 3. Downgrading due to Indirectness; 4. Downgrading due to Imprecision; 5. Downgrading due to Publication Bias; 6. Downgrading due to Intransitivity (for indirect evidence); 7. Downgrading due to Incoherence (for mixed estimates).

# Table 8 GRADE Assessment for Bristol score

| **Comparison** | **Direct evidence** | | **Indirect evidence** | | **Network meta-analysis** | |
| --- | --- | --- | --- | --- | --- | --- |
|  | **Risk ratio**  **(95%CrI)** | **Quality of**  **evidence** | **Risk ratio**  **(95%CrI)** | **Quality of**  **evidence** | **Risk ratio**  **(95%CrI)** | **Quality of**  **evidence** |
| MP vs EA | 0.095 (−0.46, 0.66) | High | 1.8 (0.73, 2.9) | High | 0.47 (−0.28, 1.2) | High |
| Placebo vs EA | −0.31 (−0.79, 0.17) | High | −2.0 (−3.1, −0.88) | High | −0.59 (−1.3, 0.089) | High |
| Placebo vs MP | −2.1 (−3.1, −1.2) | High | −0.41 (−1.2, 0.33) | High | −1.1 (−1.9, −0.17) | High |
| LT vs ACE | −0.45 (−1.4, 0.55) | High | — | — | −0.44 (−1.9, 1.1) | High |
| LT vs CHD | −0.10 (−1.1, 0.86) | High | — | — | −0.10 (−1.6, 1.4) | High |
| MP vs CHD | −1.5 (−2.2, −0.79) | Low^1,2^ | — | — | −1.5 (−2.5, −0.40) | Low |
| Placebo vs CSBS | −0.56 (−1.5, 0.43) | High | — | — | −0.56 (−2.1, 0.95) | High |
| LT_PB vs LT | 0.46 (−0.24, 1.2) | Low^2,5^ | — | — | 0.46 (−0.61, 1.5) | Low |
| PEG vs LT | 0.43 (−0.48, 1.3) | High | — | — | 0.44 (−1.0, 1.9) | High |
| MP_CHD vs MP | 1.3 (0.28, 2.3) | Very low^1,3,5^ | — | — | 1.3 (−0.24, 2.8) | Very low |
| RT vs Placebo | 1.2 (0.20, 2.2) | High | — | — | 1.2 (−0.30, 2.7) | High |
| ACE vs CHD | — | — | 0.35 (-1.78, 2.53) | High |  | High |
| ACE vs CSBS | — | — | 2.32 (-0.63, 5.24) | Low^6^ |  | Low |
| ACE vs EA | — | — | 2.29 (-0.2, 4.82) | Low^6^ |  | Low |
| ACE vs LT+PB | — | — | 0.45 (-1.04, 1.98) | Low |  | Low |
| ACE vs MP | — | — | -0.01 (-1.81, 1.85) | Moderate |  | Moderate |
| ACE vs MP+CHD | — | — | 1.82 (-0.55, 4.21) | Very low |  | Very low |
| ACE vs PEG | — | — | 0.53 (-2.27, 3.36) | High |  | High |
| ACE vs Placebo | — | — | 0.02 (-2.08, 2.12) | Low^6^ |  | Low |
| ACE vs RT | — | — | 2.87 (0.3, 5.4) | Low^6^ |  | Low |
| CHD vs CSBS | — | — | 1.96 (-0.1, 4) | Low^6^ |  | Low |
| CHD vs EA | — | — | 1.93 (0.64, 3.21) | Moderate |  | Moderate |
| CHD vs LT+PB | — | — | 0.1 (-1.43, 1.58) | Moderate |  | Moderate |
| CHD vs MP+CHD | — | — | -0.36 (-2.19, 1.45) | Very low |  | Very low |
| CHD vs PEG | — | — | 1.46 (0.42, 2.51) | High |  | High |
| CHD vs Placebo | — | — | 0.17 (-1.67, 2.02) | Moderate |  | Moderate |
| CHD vs RT | — | — | -0.34 (-2.44, 1.77) | Low^6^ |  | Low |
| CSBS vs EA | — | — | -0.03 (-1.67, 1.65) | High |  | High |
| CSBS vs LT | — | — | -1.86 (-4.35, 0.7) | Low^6^ |  | Low |
| CSBS vs LT+PB | — | — | -2.32 (-5.03, 0.44) | Low^6^ |  | Low |
| CSBS vs MP | — | — | -0.49 (-2.26, 1.29) | High |  | High |
| CSBS vs MP+CHD | — | — | -1.79 (-4.06, 0.58) | Very low^6^ |  | Very low |
| CSBS vs PEG | — | — | -2.3 (-5.21, 0.66) | Moderate^6^ |  | Moderate |
| CSBS vs RT | — | — | 0.56 (-0.95, 2.07) | High |  | High |
| EA vs LT | — | — | -1.83 (-3.82, 0.17) | Moderate |  | Moderate |
| EA vs LT+PB | — | — | -2.3 (-4.54, -0.06) | Low^6^ |  | Low |
| EA vs MP+CHD | — | — | -0.47 (-1.22, 0.3) | Low |  | Low |
| EA vs PEG | — | — | -1.76 (-3.47, -0.07) | Moderate^6^ |  | Moderate |
| EA vs RT | — | — | -2.27 (-4.74, 0.21) | High |  | High |
| LT vs MP | — | — | 1.37 (-0.49, 3.2) | Moderate |  | Moderate |
| LT vs MP+CHD | — | — | 0.08 (-2.32, 2.46) | Low |  | Low |
| LT vs Placebo | — | — | -0.43 (-1.92, 1.04) | Low^6^ |  | Low |
| LT vs RT | — | — | 2.41 (0.37, 4.44) | Low^6^ |  | Low |
| LT+PB vs MP | — | — | 1.82 (-0.27, 3.96) | Low |  | Low |
| LT+PB vs MP+CHD | — | — | 0.53 (-2.06, 3.17) | Low^6^ |  | Low |
| LT+PB vs PEG | — | — | 0.02 (-1.76, 1.84) | Moderate |  | Moderate |
| LT+PB vs Placebo | — | — | 2.88 (0.6, 5.17) | Low^6^ |  | Low |
| LT+PB vs RT | — | — | 1.66 (-1.12, 4.43) | Low^6^ |  | Low |
| MP vs PEG | — | — | -1.8 (-4.15, 0.55) | Moderate |  | Moderate |
| MP vs RT | — | — | 1.05 (0.14, 1.94) | High |  | High |
| MP+CHD vs PEG | — | — | -0.51 (-3.32, 2.26) | Moderate |  | Moderate |
| MP+CHD vs Placebo | — | — | 2.35 (0.58, 4.08) | Moderate |  | Moderate |
| MP+CHD vs RT | — | — | 1.12 (-1.22, 3.45) | Low^6^ |  | Low |
| PEG vs Placebo | — | — | 2.85 (0.29, 5.35) | Moderate^6^ |  | Moderate |
| PEG vs RT | — | — | 1.65 (-1.37, 4.55) | Moderate^6^ |  | Moderate |

**GRADE Assessment:** Reasons for downgrading evidence (1 to 7): 1. Downgrading due to Risk of Bias; 2. Downgrading due to Inconsistency; 3. Downgrading due to Indirectness; 4. Downgrading due to Imprecision; 5. Downgrading due to Publication Bias; 6. Downgrading due to Intransitivity (for indirect evidence); 7. Downgrading due to Incoherence (for mixed estimates).
