# Supplementary figures and images for: Comparative efficacy of pharmacological and nonpharmacological treatments for chronic idiopathic constipation in China: a Bayesian network meta-analysis
Source: BMC Complement Altern Med. 2019 Nov 14;19:311. doi: 10.1186/s12906-019-2741-z (PMC6857160; doi:10.1186/s12906-019-2741-z)

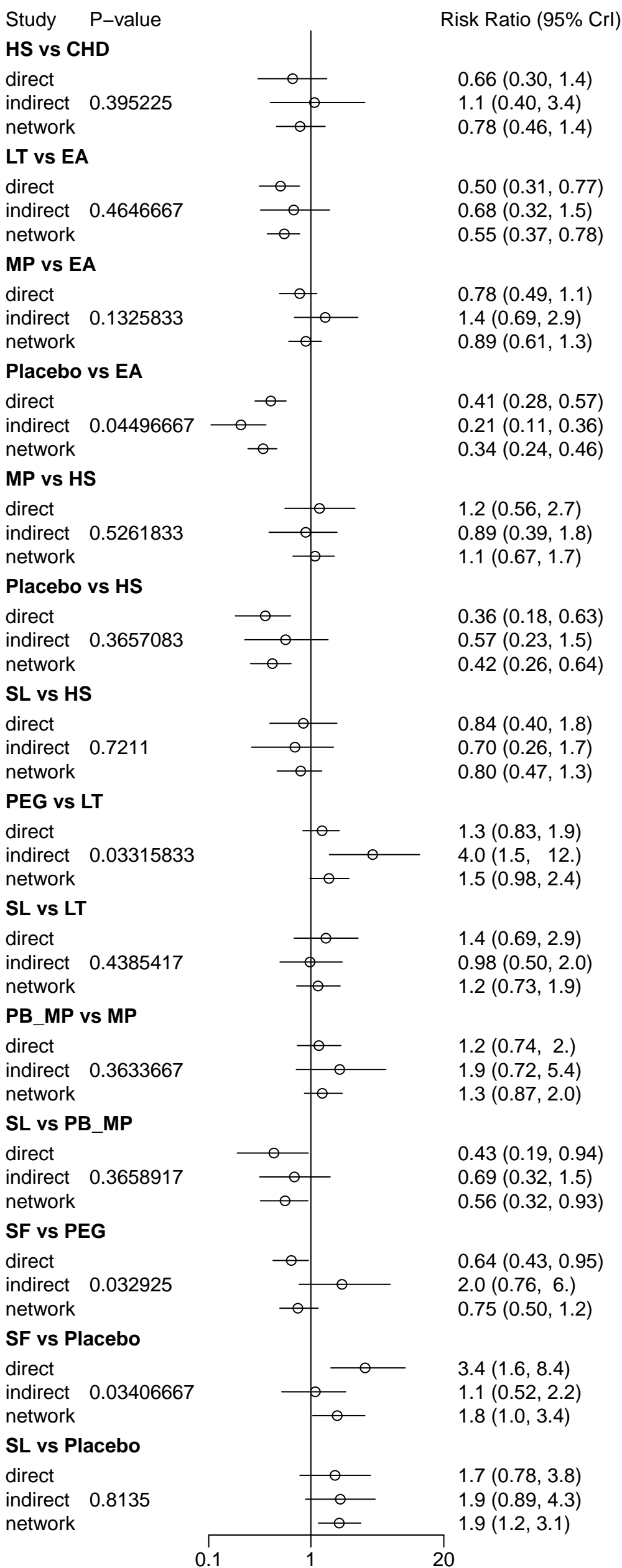

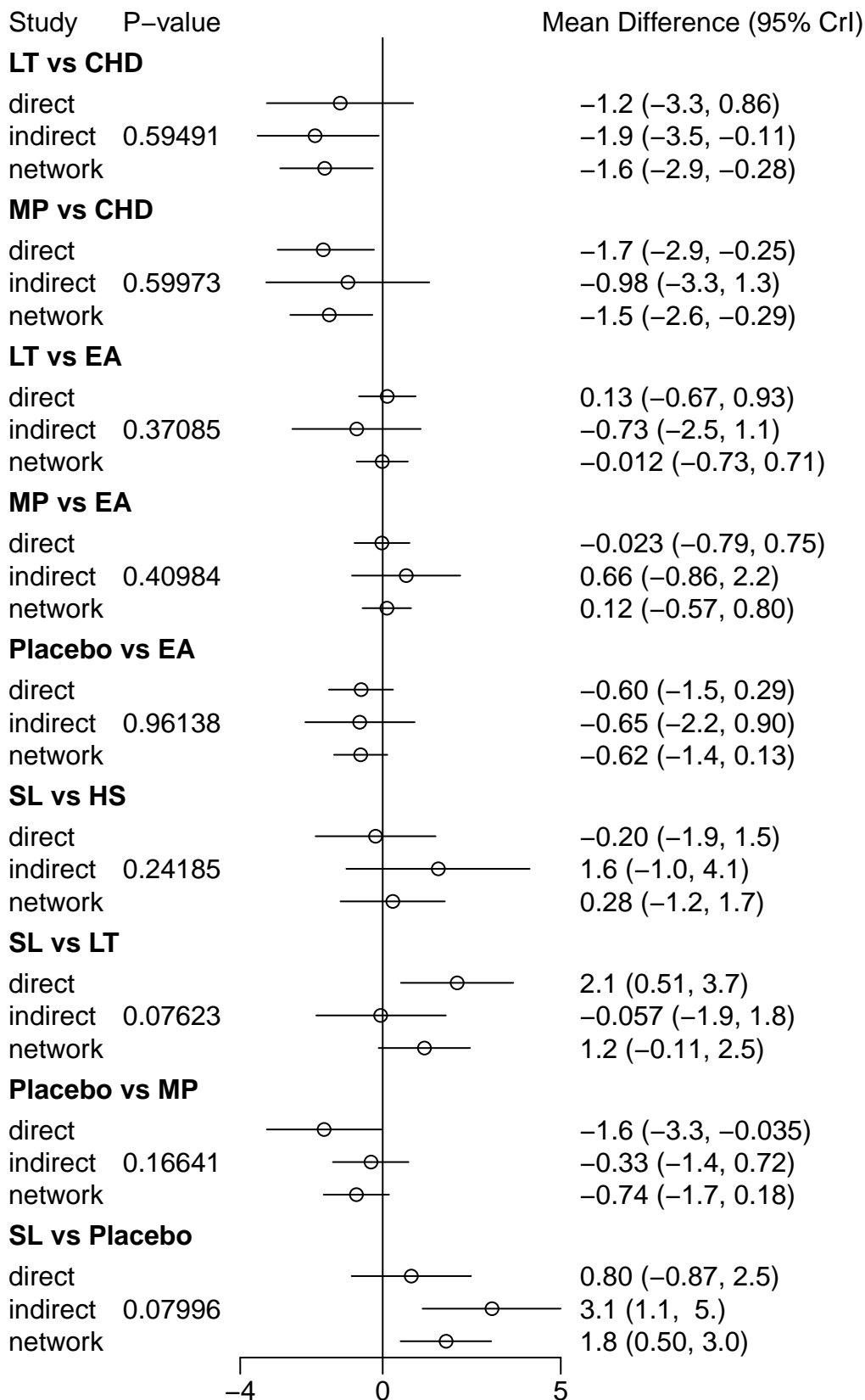

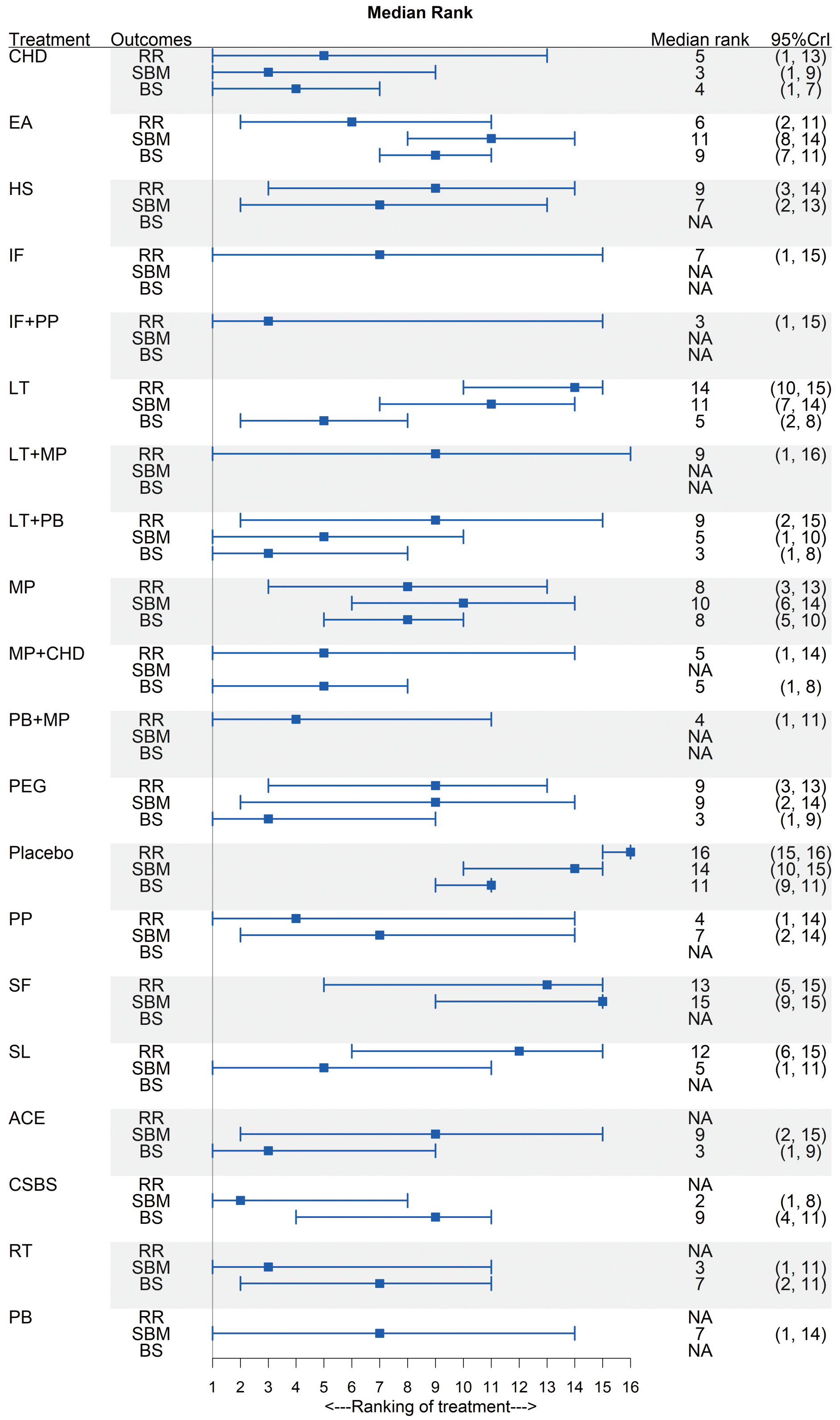

Supplement: Supplementary file 2 — Additional file 2: Page S1. Node-splitting analysis for primary outcome. Page S2. Node-splitting analysis for spontaneous bowl movements. Page S3. Forest plot of median ranks for summarized three outcomes. [file 12906_2019_2741_MOESM2_ESM.pdf]
